# Measuring intracranial pressure by invasive, less invasive or non-invasive means: limitations and avenues for improvement

**DOI:** 10.1186/s12987-020-00195-3

**Published:** 2020-05-06

**Authors:** Karen Brastad Evensen, Per Kristian Eide

**Affiliations:** 1 Department of Neurosurgery, Oslo University Hospital-Rikshospitalet, P.O. Box 4950, Nydalen, 0424 Oslo, Norway; 2 Department of Informatics, Faculty of Mathematics and Natural Sciences, University of Oslo, Oslo, Norway; 3 Institute of Clinical Medicine, Faculty of Medicine, University of Oslo, Oslo, Norway

**Keywords:** Intracranial pressure, Non-invasive ICP, Static ICP, Pulsatile ICP, Miniature pressure sensors

## Abstract

Sixty years have passed since neurosurgeon Nils Lundberg presented his thesis about intracranial pressure (ICP) monitoring, which represents a milestone for its clinical introduction. Monitoring of ICP has since become a clinical routine worldwide, and today represents a cornerstone in surveillance of patients with acute brain injury or disease, and a diagnostic of individuals with chronic neurological disease. There is, however, controversy regarding indications, clinical usefulness and the clinical role of the various ICP scores. In this paper, we critically review limitations and weaknesses with the current ICP measurement approaches for invasive, less invasive and non-invasive ICP monitoring. While risk related to the invasiveness of ICP monitoring is extensively covered in the literature, we highlight other limitations in current ICP measurement technologies, including limited ICP source signal quality control, shifts and drifts in zero pressure reference level, affecting mean ICP scores and mean ICP-derived indices. Control of the quality of the ICP source signal is particularly important for non-invasive and less invasive ICP measurements. We conclude that we need more focus on mitigation of the current limitations of today’s ICP modalities if we are to improve the clinical utility of ICP monitoring.

## Measurement of intracranial pressure (ICP)

Many consider continuous intracranial pressure (ICP) monitoring a cornerstone in surveillance and diagnostics of neurological and neurosurgical patients. However, some aspects of ICP monitoring remain controversial, including the clinical indications for ICP, the role of ICP in predicting clinical outcome of disease or treatment protocols, the clinical utility of the various ICP metrics, and how ICP should best be measured. The debate continues even though today’s clinical practice has evolved over six decades.

### The pathophysiological rationale for measuring ICP

The pathophysiological rationale for measuring ICP relies on the fact that the skull is solid, thereby restricting expansion of the total volume of the intracranial content. The main constituents of the intracranial compartment are the parenchyma of the central nervous system (CNS), cerebrospinal fluid (CSF) and blood (arterial/venous). According to the Monro-Kellie doctrine, the total volume of parenchyma, CSF and blood are constant [1]. Hence, an increase in volume in any of these components or other expansions (e.g. bleeds, tumor) must be compensated for by a reduction in the volume of parenchyma, CSF and/or blood. Measurement of ICP is used to assess the consequences of intracranial volume changes on the intracranial condition.

Intracranial pathology such as mass lesions from traumatic brain injury (TBI) may create pressure gradients [2]. The pressure gradients may result in herniation of brain tissue relative to the meninges, which again can cause compromised blood circulation or damage due to direct pressure on central nervous structures. For example, pressure to the midbrain or brainstem may have life-threatening effects on vital functions (respiratory and cardiovascular failure) and consciousness. Accordingly, one reason for ICP monitoring is to prevent the consequences of brain herniation.

Any intracranial disease process or expansive lesion with the potential to create an intracranial volume expansion also has the potential to affect energy supply to the brain through compromised blood supply to the CNS. Energy to the brain and CNS is delivered by arterial blood flow, which provides oxygen/glucose that is required for cell metabolism. Any mechanism compromising the properties and state of the intracranial compartment, hampering cerebral blood flow (CBF), represents a threat to CNS function. Therefore, the prevention of compromised CBF is one main reason for measuring ICP [3]. In neuro-intensive care, the main concern is to prevent high ICP to secure sufficient energy supply to the brain cells [4]. The cerebral perfusion pressure (CPP) is the parameter most extensively applied for this purpose, which refers to the difference between mean arterial blood pressure (BP) and mean ICP (mean CPP = mean arterial BP–mean ICP). Measurements of ICP and the ICP-derived score, CPP, is the main clinical approach for assessing compromised CBF [5]. In neuro-intensive care, CPP-oriented management is used most widely throughout the world, although alternative approaches such as variants of the Lund concept are used in some institutions [6].

It should be noted that physiological variables such as CBF, brain oxygenation and cerebral energy transfer are regulated by complex mechanisms beyond ICP regulation. It is well established that the CBF is heavily impacted by the cerebral autoregulatory capacity and influence of hyper-/hypocapnia, which controls the cerebrovascular resistance [7]. Brain oxygenation is altered in hypoxia and ischemia and depends on the state of the cerebral microcirculation. The relationship between ICP and brain oxygenation is poorly understood. In children with severe TBI, no association between ICP and partial pressure of brain tissue oxygen (PbtO_2_) and no upper critical ICP threshold for low PbtO_2_ was found [8]. The brain energy transfer also depends on various factors such as glucose availability and utilization and mitochondrial function [9]. In patients with idiopathic normal pressure hydrocephalus (iNPH), there was a significant positive correlation between fraction of sick mitochondria in perivascular astrocytic endfeet and pulsatile ICP scores [10].

**Fig. 1:**
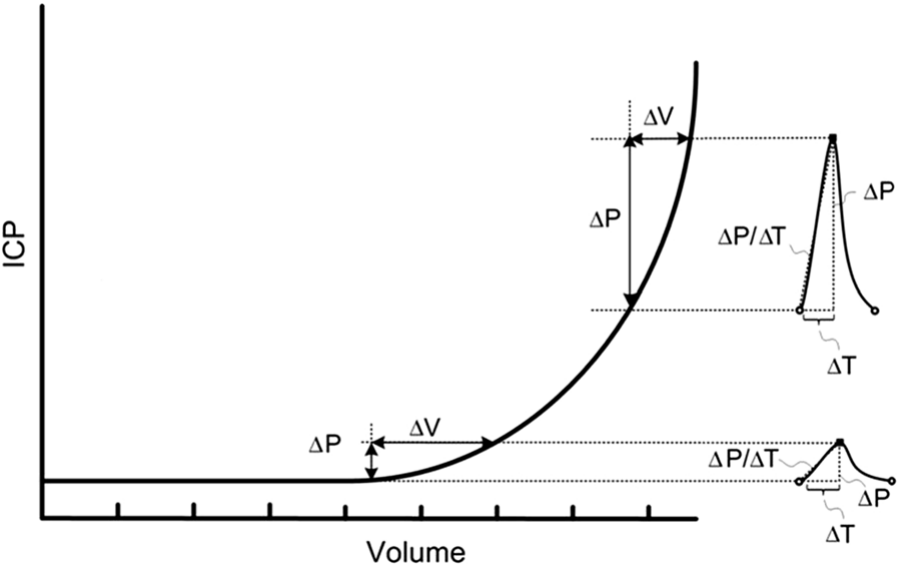
The intracranial pressure–volume curve. There is a non-linear relationship between change in intracranial pressure (ICP) and intracranial volume (Volume). At the flat portion of the curve, the pressure–volume reserve or buffering capacity is good (i.e. the intracranial compartment accepts a rather large change in intracranial volume without resulting in increased ICP). This implies that intracranial elastance is low (intracranial compliance is high). At the vertical portion of the curve, a small change in intracranial volume causes a marked rise in ICP; pressure–volume reserve capacity is low (high intracranial elastance or low intracranial compliance). The pressure–volume curve was established from measuring mean ICP. In the context of pulsatile ICP, at the flat portion of the curve the net intracranial blood volume change during the cardiac beat (about 1 ml) causes a small single wave amplitude (< 3–4 mmHg). At the vertical portion of the curve, the same net intracranial blood volume change during the cardiac beat (about 1 ml) results in a much larger ICP wave amplitude (> 4–5 mmHg). From Wagshul et al. [32]

Another important aspect of measuring ICP is securing the pressure–volume buffering or reserve capacity, which is more commonly referred to as intracranial compliance (ICC). The pressure–volume curve (Fig. 1) describes the relationship between change in ICP and change in volume of the intracranial constituents (e.g. blood, CSF or a mass). The relationship between change in pressure and change in volume is denoted as intracranial elastance (ICE; ICE = dP/dV) and is the inverse of intracranial compliance (ICC; ICC = 1/ICE). Accordingly, the term intracranial compliance or ICC refers to the capacity of the intracranial constituents to compensate for changes in intracranial volume. A more detailed discussion about ICC is given in the "Intracranial compliance (ICC)" section.

### ICP measurements in a historical perspective

In 1891, Heinrich Quincke [11] was the first to indirectly measure ICP, or lumbar cerebrospinal fluid (CSF) pressure, via lumbar puncture, but it took more than half a century before ICP monitoring was introduced in clinical practice. The year 2020 marks 70 years since Pierre Janny, in France, published his thesis on ICP monitoring [12] and 60 years since Nils Lundberg, in Sweden, presented his [13]. Lundberg’s work on measurements of ICP via ventricular puncture was instrumental in establishing ICP as a clinical tool. Many still consider measuring ICP using fluid-filled systems via ventricular catheters as the gold standard.

A major technical advancement was the introduction of dedicated ICP micro transducers in the 1980s [14]. The dedicated ICP sensors were built using different methodologies, including fiber-optic technology, strain gauge, and pneumatic principles. They were analog devices that either stood alone or were connected to vital signs monitors. Even today, the ICP field has entered the digital era to only a limited degree. Digital systems and software for ICP monitoring are still considered research tools and have been introduced in clinical routines in only a few institutions. ICP monitoring equipment for digital handling of ICP signals is expected to be introduced in the years to come.

Since measurements of ICP are invasive, because they require neurosurgical expertise, and have an inherent risk of complications, researchers have explored various less invasive and non-invasive approaches to ICP monitoring. The term non-invasive ICP (nICP) refers to the use of a non-invasive source signal for ICP estimation. Even though the field of nICP monitoring has been discouraging, the search for new nICP methodologies continues to attract the interest of researchers. However, the current clinical practice of ICP measurement relies on invasive measurements.

### Today’s practice of measuring ICP

Today, the clinical practice of measuring ICP varies greatly between centers throughout the world, but some common trends can be identified.

The main area for ICP monitoring is the surveillance of individuals treated within the neuro-intensive care unit. Many consider this modality a cornerstone in the monitoring of critically ill patients within these units [4]. In a broad sense, the patients who receive ICP monitoring can be subdivided into three categories. First, and most common, are individuals with TBI [15, 16] where the average ICP in the first 48 h after TBI was found to be an independent predictor of mortality and functional outcome after 6 months [17]. Second, ICP has a place in neuro-intensive surveillance of non-TBI patients such as patients suffering from cerebral bleeds, including subarachnoid hemorrhage (SAH) and spontaneous intracerebral bleeds with mass effect, and central nervous system (CNS) infections [18–22]. Non-TBI surveillance may also include systemic diseases such as acute liver failure, end-stage kidney failure, and hypertensive encephalopathy [23]. Third, in some centers, measurement of ICP is used for diagnostics of sub-acute or chronic health issues related to CSF disturbances, including diagnostics of patients with hydrocephalus (communicating hydrocephalus, idiopathic normal pressure hydrocephalus), idiopathic intracranial hypertension, and Chiari malformation [24–26]. Some centers have also implemented indirect measurement of ICP by lumbar infusion tests in conjunction with assessment of resistance to CSF outflow as clinical routine [27].

Current measurements of ICP most commonly include a pressure transducer from either a fluid-filled system or a dedicated system that can be connected to a vital signs monitor capable of presenting the ICP as numerical values of mean ICP. The mean ICP score refers to the absolute or static ICP relative to a reference. Some systems allow for the presentation of trend plots of numerical values. The trend plots present mean ICP over time, with variable update frequency (often one value every 30s or minute). An early attempt to assess the burden of intracranial hypertension [28] was by means of analysis of frequency or weight of certain mean ICP levels.

**Fig. 2:**
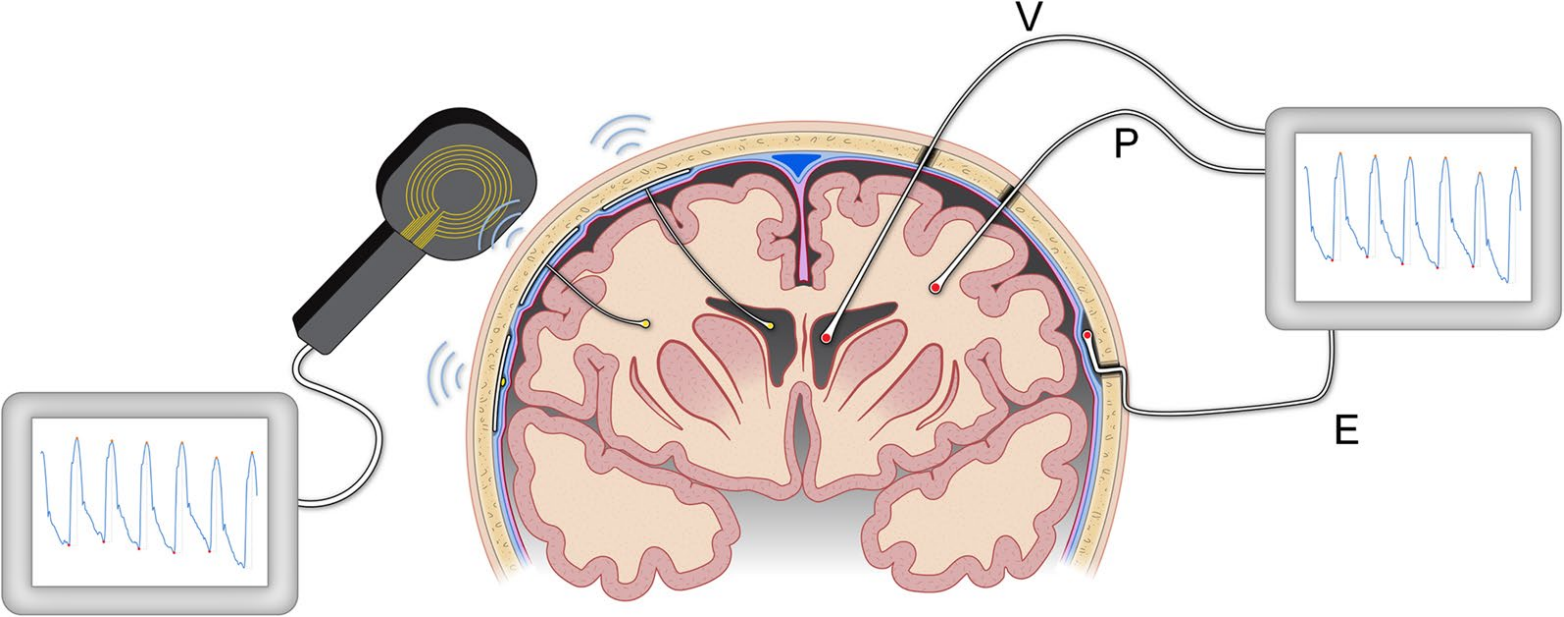
Overview of wire-based and wireless methods for ICP monitoring. The image on the right shows that ICP is measured via a ventricular (V) catheter placed within the cerebral ventricles, and dedicated ICP sensors implanted within the brain parenchyma (P), or via the ICP sensor placed within the epidural (E) location. The invasive ICP source signals are transferred to a monitor that may reveal the ICP scores. For example, the ICP scores may be shown as numerical values, trend plots, or as the single ICP waves. The image on the left illustrates implantable sensors to the ventricles or parenchyma wherein the communication between sensor and external receiver is wireless. Illustration: Øystein Horgmo, University of Oslo

Measuring ICP via a CSF ventricular catheter is still the most widespread approach, even though the use of dedicated ICP sensors placed in the brain parenchyma has become more common since the 1980s [14] (Fig. 2). Epidural ICP measurements, referring to the placement of the sensor between the skull and the dura mater, are generally no longer used, as this method was found to be inaccurate, although a few centers have reported epidural ICP [29] as clinically useful.

In recent years, implantable ICP sensors (i.e. telemetric ICP sensors) have been introduced on the market, and some early experience has been reported [30, 31]. The telemetric systems provide the opportunity to implant dedicated ICP sensors, enabling assessment of mean ICP from an external receiver (Fig. 2). This latter approach may be advantageous in individuals with CSF disturbance and in individuals with suspected shunt failure.

**Fig. 3:**
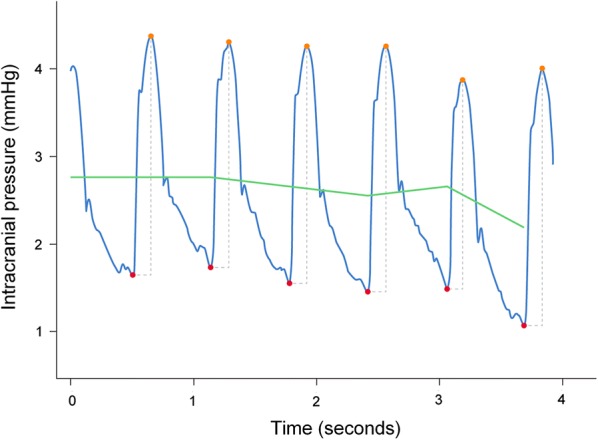
Measurements of static versus pulsatile ICP. The static ICP (mean ICP) is an absolute pressure value measured against a reference pressure (here illustrated by the green line), not considering the pressure changes occurring during the cardiac cycle. The pulsatile ICP is the pulse pressure or the pressure changes occurring during the cardiac cycle (here illustrated by the blue line). A single ICP wave is characterized by an increase in pressure from diastolic minimum pressure to systolic maximum pressure (the peaks are illustrated by the red dots). The single ICP wave amplitude is the peak-to-peak pressure difference. Illustration: Øystein Horgmo, University of Oslo

Today’s clinical practice of ICP monitoring utilizes mean ICP (static pressure) extensively. The literature regarding the clinical usefulness of ICP-derived scores other than the mean, however, is growing [32]. The most established include various approaches to pulsatile ICP monitoring, which refers to the pressure changes that occur during each cardiac cycle and is usually denoted as the pulse pressure or single ICP wave pressure (Fig. 3). Currently, pulsatile ICP analysis is performed in only a limited number of centers.

### The clinical value of ICP monitoring

Despite the 60–70 year history of ICP monitoring and significant improvements in ICP measurement technology, its role continues to be a matter of debate. For example, this controversy is evident within the field of TBI [15, 33–35], which is the clinical field that has provided the largest amount of knowledge about ICP. The lack of consensus is further illustrated by the substantial variation in clinical indications for ICP monitoring between centers [36]. In addition, the guidelines for management of severe TBI acknowledge that the widespread use of ICP monitoring has limited support in existing ICP literature [16] as Class 1 evidence for the clinical use of ICP monitoring is lacking. A randomized controlled trial (RCT) found no improved outcome in individuals with TBI managed according to ICP monitoring that aimed to keep the pressure below 20 mmHg, compared with a group of individuals not undergoing ICP monitoring as part of surveillance [35]. However, one criticism against the study protocol is that it compared two different treatment approaches rather than assessing the value of ICP monitoring per se [37].

One criticism is that comparisons between scientific studies are difficult or impossible since the ICP measurement methodologies are standardized to a limited degree. However, it may be argued that the role of ICP in intracranial pathophysiology has rather strong support.

### ICP versus other physiological parameters: multi-modality monitoring

Intracranial disease processes and pathophysiological events after e.g. injuries or bleeds, involve complex cascades of events beyond alterations in ICP. The ICP primarily refers to the pressure and pressure–volume reserve within the skull, and therefore describes only part of the pathophysiological cascades. For this reason, monitoring of several physiological variables (e.g. CBF, brain oxygenation and metabolism), referred to as multi-modality monitoring, has been advocated [9, 38]. Through this type of monitoring, ICP can be measured along with other parameters such as brain tissue oxygenation (PbtO_2_), temperature, cerebral blood flow velocity, cerebral metabolism (micro-dialysis), and assessment of electro cortical activity. The multi-modality approach allows for incorporation of different aspects of the brain state in patient surveillance and can contribute to a more holistic view of the patient’s condition. Catheters for multi-parametric measurements, intended for neuro-intensive care, have therefore appeared on the market. The Neurovent-PTO (Raumedic) is one example of an integrated catheter that allows for simultaneous measurements of ICP, temperature and tissue oxygen (O_2_) [39].

In this review, we focus on ICP, because this variable is by far the most applied monitoring parameter while several of the other monitoring modalities currently are tools for research and have not been adapted to clinical practice. We would like to stress, however, that limitations and weaknesses of individual modalities may not be overcome by monitoring many physiological parameters. Continuing the search for limitations associated with the individual modalities is therefore warranted so as to provide the best possible patient care.

## Invasive ICP source signals

Irrespective of the ICP or ICP-derived score presented to the doctor or nurse, the clinical value depends on the quality of the measured input signal. Most commonly, ICP is measured directly by invasive placement of a pressure probe in the intraventricular and CNS parenchyma locations [4]. The subdural location represents an alternative probe placement location, but is usually only applied as part of a craniotomy [40].

### ICP measurements from fluid-filled ventricular catheters

Measuring ICP via a CSF ventricular drain was the initial ICP monitoring approach and is still considered by many to be the gold standard. Under this measurement protocol, a fluid-filled drain is connected to an external fluid pressure sensor providing an opportunity for therapeutic CSF drainage. However, the procedure requires neurosurgical expertise to open the skull, penetrate the dura and introduce a catheter through the brain parenchyma, and then insert the tip of the catheter into one of the lateral ventricles. For accurate pressure level monitoring, the external pressure transducer must be zeroed correctly towards ambient pressure. Frequent zeroing to ensure valid ICP measurements and remove potential drift is considered good protocol if the ruling aseptic protocols are followed.

The use of ventricular catheters has been advocated in particular, due to intracranial pressure-gradients, which will cause placement-dependent pressure readings for parenchymal probes. Measuring ICP within the CSF spaces can therefore be argued to be more reliable than parenchymal placement as intraventricular measurements will yield a universal intracranial pressure.

### Limitations

Although fluid-based ICP measurements are considered the most accurate, this measurement modality has several limitations including: (1) complication profile related to its invasive nature, (2) defining zero levels for fluid-filled catheters, and (3) inaccuracy related to ICP source signals from the fluid-filled system.

It is well established that placement of ventricular catheters represents a risk in terms of “misplacement” of catheters in the ventricles, intracerebral hemorrhages, and severe infections [41–43]. The infection rate increases with prolonged monitoring time. In some cases, placing the catheter may be difficult when ventricles are small, as in brain edema, rendering correct placement precarious. In a study from 2009, it was reported that as many as 12.3% of the catheters were misplaced [44]. From the perspective of complications, it could be argued that ICP monitoring via ventricular catheters should be used only when CSF drainage is justified. Placing a catheter in the ventricles solely for the purpose of monitoring ICP may be considered too invasive to be justifiable.

When fluid catheters are used, a zero level reference pressure must be selected; the standard protocol varies among institutions. Most commonly, the foramen of Monro is used as zero level. The different practices thereby make it difficult to directly compare ICP values measured at different institutions, which represents a challenge in clinical research. For day-to-day patient care, however, the most important is that each institution has strict definitions of the level of zero pressure when fluid-filled systems are used. However, an obvious advantage compared to other invasive measurement modalities is that the zero pressure level may be controlled and recalibrated at any time.

We do, however, wish to stress that the challenges related to measuring ICP from a fluid-filled system should not be underestimated [45]. The ICP source signal is easily corrupted by air bubbles in the catheter, or by debris causing occlusion or partial occlusion of the catheter. Since the pressure sensor is external to the patient, the distance from the site of measurement (cerebral ventricles) to the sensor is usually long. Movement of the catheter will also result in a noisy signal. Quality control is limited to visual operator-dependent and subjective inspection of the ICP waveform on the monitor screen. False measurements of ICP via ventricular catheters may be caused by blockade of the ports of the drain resulting in increased resistance to CSF flow [46].

### Dedicated implantable ICP sensors

The dedicated ICP sensors and transducers that were introduced for clinical use in the 1980s underwent thorough clinical assessment. They have since been shown to be reliable and accurate in the clinical setting. Historically, the commercial ICP sensors most extensively used include the fiber-optic Camino ICP sensor (Integra LifeSciences, Plainsboro, NJ, USA) [47, 48]; the strain gauge Codman microsensor (Codman and Shurtleff Inc., Raynham, MA, USA) [49, 50]; Raumedic Neurovent P (Raumedic AG, Helmbrechts, Germany) [51, 52]; Pressio ICP sensor (Sophysa, Orsay, France) [53]; and the pneumatic (air-pouch) Spiegelberg ICP sensor (Spiegelberg GmbH & Co KG, Hamburg, Germany) [54, 55]. In contrast to the other ICP sensors, the Spiegelberg ICP sensor incorporates a pneumatic system that is not useful for ICP waveform analysis [56].

We consider the dedicated ICP sensors to be more useful than measuring ICP via ventricular drainage systems since ventricular puncture represents a more invasive procedure with higher risk of severe complications, and the pressure signals obtained from dedicated ICP sensors are less prone to artifacts than those from a fluid-filled system. The validity of parenchymal measurements has been thoroughly documented through numerous validation studies [14, 57, 58]. The combination of these two factors makes dedicated ICP sensors a good alternative to ventricular catheters. The introduction of dedicated ICP sensors, therefore, represents a major advantage for ICP monitoring. In addition, because ventricular catheters are less safe than parenchymal probes, there are no apparent reasons for choosing this measurement protocol unless the need for CSF drainage is a given.

### Limitations

Similar to ventricular catheter placement, the insertion of dedicated ICP sensors represents a risk for bleeds and infections [14, 59, 60]. The procedure requires neurosurgical expertise. Typically, a burr hole is made in the skull and a transducer is inserted into the brain parenchyma. A non-eloquent region of the brain, such as a frontal region, is preferred. Safe placement is significantly easier with this technique compared with ventricular placement.

The duration of monitoring differs depending on whether it is performed for surveillance or for diagnostic purposes. Often, monitoring is ended after a few days due to the increased risk of infection associated with long-term placement of an intracranial device. This is despite the fact that long-term monitoring for a period of weeks to months could provide valuable information about disease development and patient rehabilitation, thus resulting in significantly better patient care.

As noted previously, the major issue with ICP sensors for measurement of mean ICP is uncontrolled alteration in zero reference pressure level because they are prone to drift, or the baseline jumps due to referencing errors [14]. These problems, however, only apply to mean ICP levels and not ICP waveforms [61]. The systems using dedicated ICP sensors placed in the parenchyma do not allow for pressure zeroing to be performed in vivo. After these pressure systems are zeroed relative to atmospheric pressure during a pre-insertion calibration, their output is dependent on zero drift of the sensor.

A criticism of the ICP sensors is that they reflect the local pressure at the site, which can be misleading as there are pressure gradients across the intracranial compartment and hence, the pressure within the skull is not uniform.

## The different ICP scores

For health care personnel in general, and for most neurosurgeons and neurologists, ICP is synonymous with a numerical value. It may be the fluid level (measured in cm H_2_O) in a drainage system, or it may be the number expressed in mmHg on the monitor screen. However, as previously noted by others [62], ICP is more than a number. A range of ICP scores and ICP-derived scores have been explored to improve clinical decision making [63]. As illustrated in Fig. 3 and Additional file 1: Movie 1, an ICP signal is a compound signal, where the static pressure value (mean ICP) only represents one aspect of the pressure signal. The ICP scores can be dichotomized as static and pulsatile ICP scores, independent of whether the ICP source signal is obtained by invasive or non-invasive means.

### Static ICP (mean ICP)

A vast amount of literature on measurement of ICP addresses the mean ICP parameter (i.e. a static or absolute pressure score). This parameter is so dominant that the term “ICP” is often used synonymously with the numerical value “mean ICP”. The mean ICP is usually expressed in mmHg, but also the units mm H_2_O and, less commonly, as Pascal (Pa) are used.

The mean ICP value is an absolute pressure value that is relative to a reference pressure value. When ICP is measured via a ventricular catheter, the reference value is the zero-value selected by the physician. Often, the foramen of Monro is defined as a zero pressure level, but the exact zero level may vary between institutions.

When ICP is measured using dedicated ICP sensors, zeroing is typically performed against atmospheric pressure and the reference value is stored in the ICP measurement system. When monitored by a medical device, the mean ICP value refers to the average of pressure values over a certain time (often 3–10 s).

Normal mean ICP values have not been established since ICP measurements in healthy individuals cannot be justified from an ethical perspective. Only indirect evidence about ICP is available from individuals that are “as normal as possible”. According to TBI guidelines, the goal is to keep mean ICP below 20 mmHg [64]. However, this threshold does not mean that a mean ICP of less than 20 mmHg would be considered normal. With regard to mean ICP, ICP measurements from subjects “as normal as possible” suggest that “normal” mean ICP is in the range of 0 to 10 mmHg [65–70] and tends to decline with increasing age [66–68]. The mean ICP scores do not seem to differ between day and night [25, 66]. On the other hand, the normal mean ICP scores are heavily contingent on body position. When a person is standing in an upright position, the mean ICP falls. Andresen et al. [71] reported that the postural difference in mean ICP could differentiate healthy individuals from patients with CSF disturbances. In our clinical practice, we consider mean ICP in an upright position of lower than − 5 mmHg as abnormal [72].

### Static ICP-derived scores

Mean ICP has been combined with other metrics to create various indices to add clinical value to mean ICP alone. By far the most well known ICP-derived index is CPP, which is the difference between mean arterial BP and mean ICP (CPP = mean arterial BP–mean ICP) [5]. Today, CPP-oriented surveillance is a cornerstone in management of both TBI and non-TBI patients, including individuals with SAH.

Other indices that are used in some centers are the RAP (correlation coefficient (R) between Amplitude and Pressure) and pressure reactivity index (PRx) indices [63, 73]. The RAP is the moving correlation between mean ICP and AMP (ICP wave amplitude), and considered to provide a measure of the pressure–volume reserve capacity [74]. A RAP > 0.6 has been interpreted as indicative of impaired pressure–volume reserve [75]. The RAP index has been referred to in numerous research papers, but its place in clinical practice remains to be clarified [76].

The PRx (moving correlation between mean ICP and mean arterial BP) is considered a measure of the cerebrovascular reactivity or a proxy of the auto-regulatory state [77], and has been used in neuro-intensive care for many years in some institutions [78, 79]. However, as for the RAP index, the clinical utility of the index is disputed [80].

The mean ICP-derived indices are not provided by commercial ICP monitoring devices, but require dedicated software.

### Limitations with static ICP scores

When addressing the limitations of current ICP measurements, the scientific literature has focused on the risks of infections and bleeds accompanying the invasiveness of the procedure. However, there are challenges related to the lack of ICP signal quality control and reference pressure variability, which are present even in standardized invasive monitoring [14, 81]. These aspects have been given limited attention, despite their significance in the clinical context.

As the possible consequences of inaccurate ICP measurements are severe, the standards for accuracy of ICP measurements and measurement devices are strict. These device standards were previously developed by The American National Standards Institute (ANSI)/Association for the Advancement of Medical Instrumentation (AAMI) [82]. With regard to ICP measurement accuracy, the ANSI/AAMI standards state that the ICP monitoring devices should provide an accuracy of ± 2 mmHg in the ICP range 0–20 mmHg. Moreover, the maximum error should not exceed ± 10% in a range of 20–100 mmHg. With regard to nICP monitoring, the AAMI also states that when ICP is between 0 and 20 mmHg, a difference of 2 mmHg is acceptable when comparing nICP and ICP measurements, and when ICP is 20–100 mmHg, the difference should be less than 10% [83].

When a patient is undergoing continuous ICP monitoring, however, health care personnel have few tools available to assess the accuracy of the ICP measurement. Often the control is limited to some form of visual inspection of a snapshot of a processed signal, or in the case of CSF drain, the fluctuations of the fluid within the drain. Few institutions apply technology that creates trend plots of different ICP scores. This makes it impossible to accurately evaluate long-term trends and generally makes the assessments unreliable and operator-dependent.

## An illustrative case

**Fig. 4:**
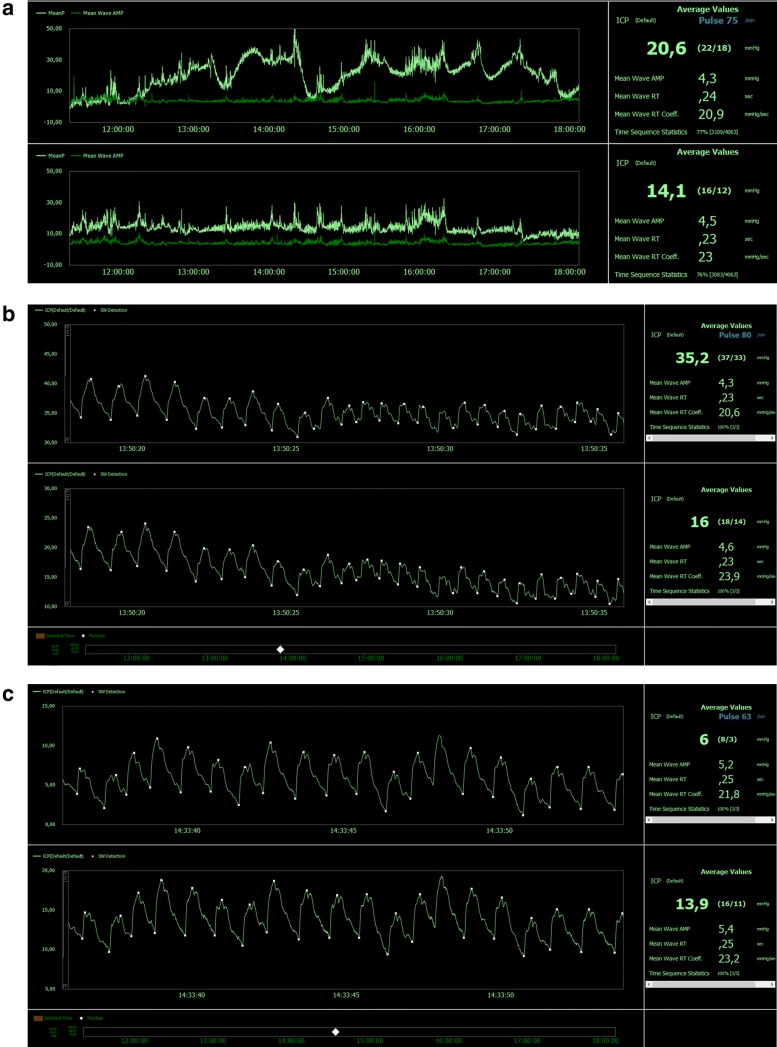
Continuous ICP measurement from an individual with subarachnoid hemorrhage. Intracranial pressure was measured from two separate ICP sensors placed nearby in the right frontal lobe of an individual suffering from a subarachnoid hemorrhage 3 days before. The left upper window (**a**) presents the trend plots of mean ICP (MeanP, light green) and mean ICP wave amplitude (MeanWave AMP, darker green) measured from a Camino ICP sensor, and the lower left window the trend plots of mean ICP (MeanP, light green) and mean ICP wave amplitude (MeanWave AMP, darker green) measured from a Codman ICP sensor. Average values from the Camino ICP sensor (upper window) are Mean ICP 20.6 mmHg, Mean Wave AMP (amplitude) 4.3 mmHg, Mean wave RT (Rise time) 0.24 s, Mean Wave RT Coeff (Rise time coefficient) 20.9 mmHg/seconds. Average values of the Codman ICP sensor (lower window) are Mean ICP 14.1 mmHg, Mean Wave AMP (amplitude) 4.5 mmHg, Mean wave RT (Rise time) 0.23 s, Mean Wave RT Coeff (Rise time coefficient) 23 mmHg/seconds. In (**b**) the ICP waveform of the Camino (left upper window) and Codman (left lower window) ICP sensors are shown. The ICP scores are presented in the right windows. Despite close to identical ICP waveform from the Camino and Codman ICP sensors, the mean ICP differed substantially (mean ICP of Camino ICP 35.2 mmHg and mean ICP of Codman 16 mmHg). Subfigure (**c**) presents the ICP waveforms at a later time point. The Camino recording is shown in the left upper window and the Codman recording in the left lower window. At this time point, the mean ICP was lower in the Camino (6.0 mmHg) than Codman ICP sensors (mean ICP 13.9 mmHg); the ICP waveforms were close to identical. The pressure recording was retrieved from a pressure quality registry at Oslo university hospital (Approval 2014/4720)

It is important to bear in mind that measurement of ICP is no treatment per se, but a monitoring modality that may aid in patient treatment. Given the importance of this modality for patient management, clinicians must be able to trust the measured ICP. Unfortunately, when ICP is measured from only one ICP sensor, it may be hard to ascertain whether the ICP is real or not. In a few cases, it has been possible to measure ICP from two separate ICP sensors placed nearby in the brain parenchyma. One such case is illustrated here (Fig. 4). This ICP recording was retrieved from a pressure quality registry at Oslo university hospital (Approval 2014/4720).

Figure 4 shows the trend plots of mean ICP and mean ICP wave amplitude (MWA) from one of our patients (Fig. 4a). This individual was hospitalized due to a subarachnoid hemorrhage, and underwent craniotomy with clipping of the aneurysm and placement of an external ventricular catheter. This particular ICP measurement from 11:21 to 18:07 (Fig. 4a) was obtained 3 days after the bleed. At this point of time, the patient had two ICP sensors from different manufacturers in the right frontal lobe (upper window refers to a Camino ICP sensor, and lower window to a Codman ICP sensor; Fig. 4a). The patient was sedated and on a respirator. The patient was awakened and taken off the respirator the day after the ICP recording, and had a positive clinical development thereafter. It should be noted that the ICP sensors were placed nearby. Therefore, no pressure gradients existed between the two sensors, and we would expect the two ICP sensors to yield similar readings. The Camino ICP sensor (in the upper window) consistently shows a markedly higher mean ICP score than the Codman ICP sensor (average values 20.6 mmHg versus 14.1 mmHg). The mean ICP wave (MWA) was, however, close to identical (4.3 mmHg vs. 4.5 mmHg) for the two recordings. It should also be stated that the mean ICP, and thereby many of the ICP derived scores, at some time points differed substantially. In Fig. 4b the ICP waveforms of the two signal are shown and the mean ICP of Camino ICP reveals 35.2 mmHg while the mean ICP of the Codman ICP sensor reads16 mmHg.To further illustrate, later in the recording, the Codman ICP shows a higher value (13.9 mmHg) than the Camino (6 mmHg) (Fig. 4c), making the uncertainties somewhat manufacturer-independent. It is worth accentuating, however, that the MWA is comparable at early (4.3 vs. 4.6 mmHg; Fig. 4b) and late time points (5.2 vs 5.4 mmHg; Fig. 4c) and for the two sensors throughout the recording. Which of the ICP scores should then be trusted? That of the Camino or that of the Codman? Which kinds of measures are offered to health care personnel to check quality control of ICP measurements? Since many apply 20 mmHg as the threshold for intervention, this patient could have been given very different treatment while being in the exact same state.

On this background, since mean ICP is measured against a reference value, the major limitation associated with this metric is an erroneous mean ICP because of variability of the reference pressure. Mean ICP scores such as CPP, RAP and PRx will be affected by reference pressure jumps. Several reasons for abnormal alterations in reference pressure can be defined.

### Impact of reference pressure variability on static ICP scores

Today’s practice of measuring ICP relies on measuring static ICP, which is an absolute pressure relative to a reference pressure, or a baseline pressure (Fig. 3). The mean ICP is the pressure difference between the inside of the skull and the reference pressure value (most commonly the atmospheric pressure). This reference pressure may be affected not only by the atmospheric pressure, but also by the inherent reference of the sensor. If the reference pressure varies for some reason, the calculated mean ICP becomes wrong. As the parenchymal probes are impossible to recalibrate after insertion, they are prone to drift as well as baseline shifts in reference pressure. The magnitude of drift has been addressed by examining zero pressure after explantation [49] and was found to be typically fairly low in average. It is nevertheless still a relevant risk factor. The role of instability in ICP sensor reference pressure causing sudden or high-magnitude temporary shifts in reference pressure has been less addressed despite being of higher clinical importance. This issue is more relevant when measuring from a dedicated ICP sensor than from a CSF ventricular drain. To date, the issues of ICP source signal control and reference pressure variability have been given limited attention in the literature [76].

### Drift in reference pressure when ICP is measured over longer time

The concern of sensor drift resulting in ICP changing over time is related to the fact that it is not possible to re-zero the dedicated ICP sensors after insertion [81]. This concern has been extensively addressed [14, 81, 84, 85] and is well established [48], but the magnitude of drift varies between studies [14, 81]. Typically, drift is examined after the sensors have been removed, which enables pressure measurement against the atmospheric pressure, and magnitude of drift is defined as the difference in pressure from the original reference value.

To provide a measure of drift, Morgalla et al. [84] described a drift index based on the following parameters derived over a 10-day measurement period: percentage of time involving a pressure change, maximum absolute pressure change, and the mean absolute pressure deviation. Different commercial ICP sensors gave different profiles for the drift index.

However, the assessment of drift says nothing about temporary changes during ongoing ICP monitoring due to other noise factors such as posture changes or electrostatic discharges, to name a few, which may also affect the reference pressure.

### Electrostatic discharges

It is well established that medical devices such as ICP monitoring systems may malfunction due to electrostatic discharges (ESDs) [86, 87]. All parts of the ICP measurement system, including sensor, cable, transducer and display, may represent potential sites of origin for baseline pressure errors (BPEs). Electrostatic discharges may cause abrupt or gradual changes in the zero reference pressure of ICP sensors. The pressure changes may be transient or cause lasting changes in the zero pressure level, which may result in erroneous mean ICP scores. In an experimental bench-test study, ESDs produced lasting alterations in zero pressure of > 10–20 mmHg, and this was seen for various types of ICP sensors [88]. Other studies also confirmed the sensitivity of ICP sensors to ESDs [89].

**Fig. 5:**
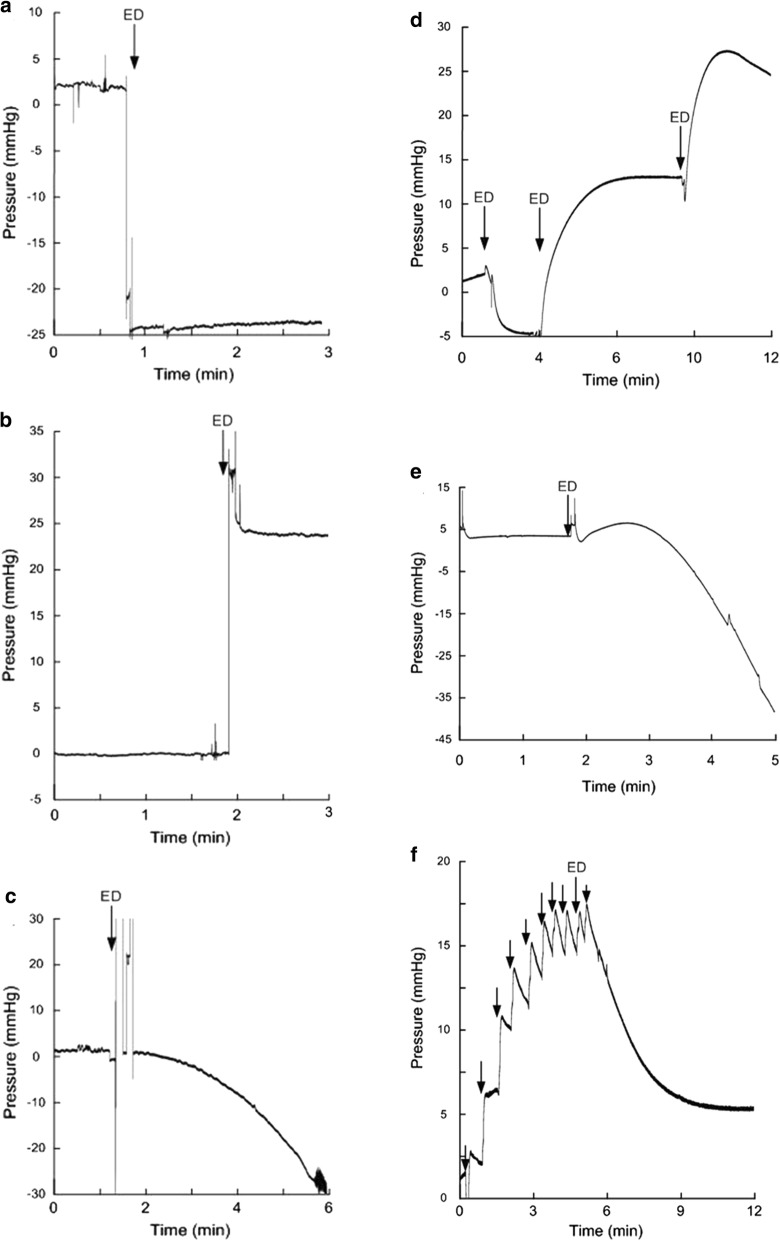
Impact of electrostatic discharges (ESDs) on mean ICP. Results from bench testing of commercial ICP sensors exposed to ESDs. The continuous pressure signal from a Codman MicroSensor is presented before and after ESD in three individuals showing (**a**) a sudden decline in ICP, (**b**) a sudden rise in ICP, and (**c**) a gradual reduction in ICP. Bench testing of a Raumedic Neurovent P sensor exposed to ESDs caused (**d**) a gradual increase in ICP, or (**e**) a gradual decline in ICP. Repeated ESDs causing a stepwise increase in ICP are shown in (**f**). The baseline pressure level (mmHg) is shown on the y-axis and time (minutes) on the x-axis. The ESD is indicated by an arrow. Notably, the ESDs were of small magnitude. When the test person was charged to 0.5 kV, the ESD delivered to the ICP sensor was typically 0.5 kV pulse peak. Charging to 5 kV gave a potential charge of 2.5 kV (2–5 kV). ESDs < 3 kV provoked few unpleasant sensations for the test person, while ESDs of about 5 kV gave unpleasant sensations. Adapted from Eide and Bakken [88]

It seems clear that major alterations in zero reference pressure due to ESDs may erroneously alter the mean ICP perceived by the health care personnel. A short-lasting change in mean ICP because of an ESD may not represent an issue. The problem arises when lasting change in mean ICP occurs (Fig. 5). In the clinical setting, a change in mean ICP due to ESD may not be detected because most ICP monitoring systems do not provide for trend plots of mean ICP. Moreover, when plotting mean ICP over time, it may be difficult to decipher whether a change in mean ICP is caused by alterations in patient state or whether it represents a technical error such as an ESD.

### Baseline pressure errors

Simultaneous measurements from two ICP sensors placed nearby can reveal markedly different mean ICP scores; Fernandez et al. [50] measured ICP from a Codman and Camino sensor and observed sudden shifts in mean ICP but could not explain this since the ICP waveform was not monitored.

**Fig. 6:**
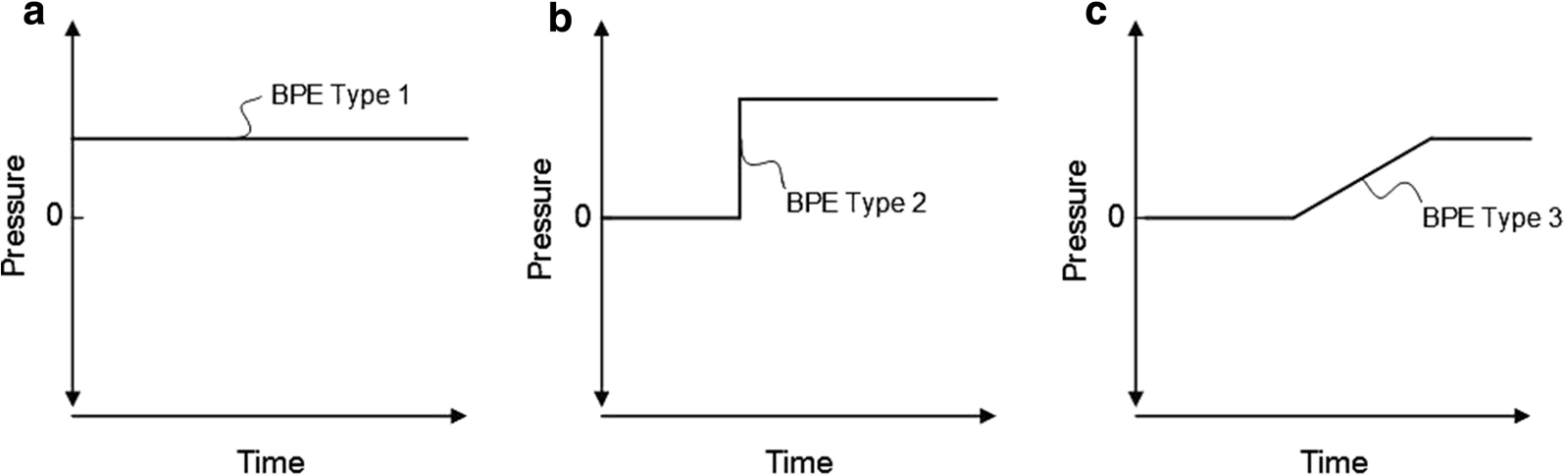
The different types of baseline pressure errors (BPEs). Graphical illustration of the different types of BPEs. **a** BPE Type 1 is characterized by a constant offset of reference pressure (e.g. due to incorrect zeroing or calibration failure). **b** BPE Type 2 is related to a sudden shift in baseline pressure. One cause may be ESDs, as illustrated in Fig. 7 a–b. **c** BPE Type 3 is related to a gradual and large magnitude change in baseline pressure. This type is typical for drift of ICP sensor reference pressure or may be caused by ESDs (see Fig. 7 c–f). Notably, these different types may occur together during ongoing ICP monitoring. From Eide et al. [92]

The term “baseline pressure errors” refers to the occurrence of marked differences in mean ICP combined with close to identical MWA and was first described when measuring continuous ICP simultaneously from two ICP sensors placed nearby within the intracranial compartment [90]. Three types of BPEs have been defined, as outlined in Fig. 6. The BPEs were seen irrespectively of type of ICP sensors and included fiber-optic (Camino), strain-gauche sensors (Codman, Raumedic Neurovent P), pneumatic (air-pouch) sensors (Spiegelberg), and fluid-based sensors (Edward’s Life Science) [45, 90]. When monitoring via fluid-filled drains, BPEs may be caused by imperfect fluid connection due to debris or air bubbles in the catheter, or even non-intentional changes in sensor position relative to the measurement site [91].

With regard to the BPE Types 1–3 illustrated in Fig. 6, Type 1 may cause a constant offset of mean ICP. This is illustrated in Additional file 3: Movie 3 where ICP measured from two separate ICP sensors placed nearby in the brain show different mean ICP scores and close to identical ICP waveforms. Moreover, BPE Type 3 is illustrated in Additional file 4: Movie 4, which illustrates how the mean ICP from only one of the ICP sensors suddenly changes while showing no change in the ICP waveform. These examples are from ICP measurements obtained from surveillance of individuals with subarachnoid hemorrhage.

**Fig. 7:**
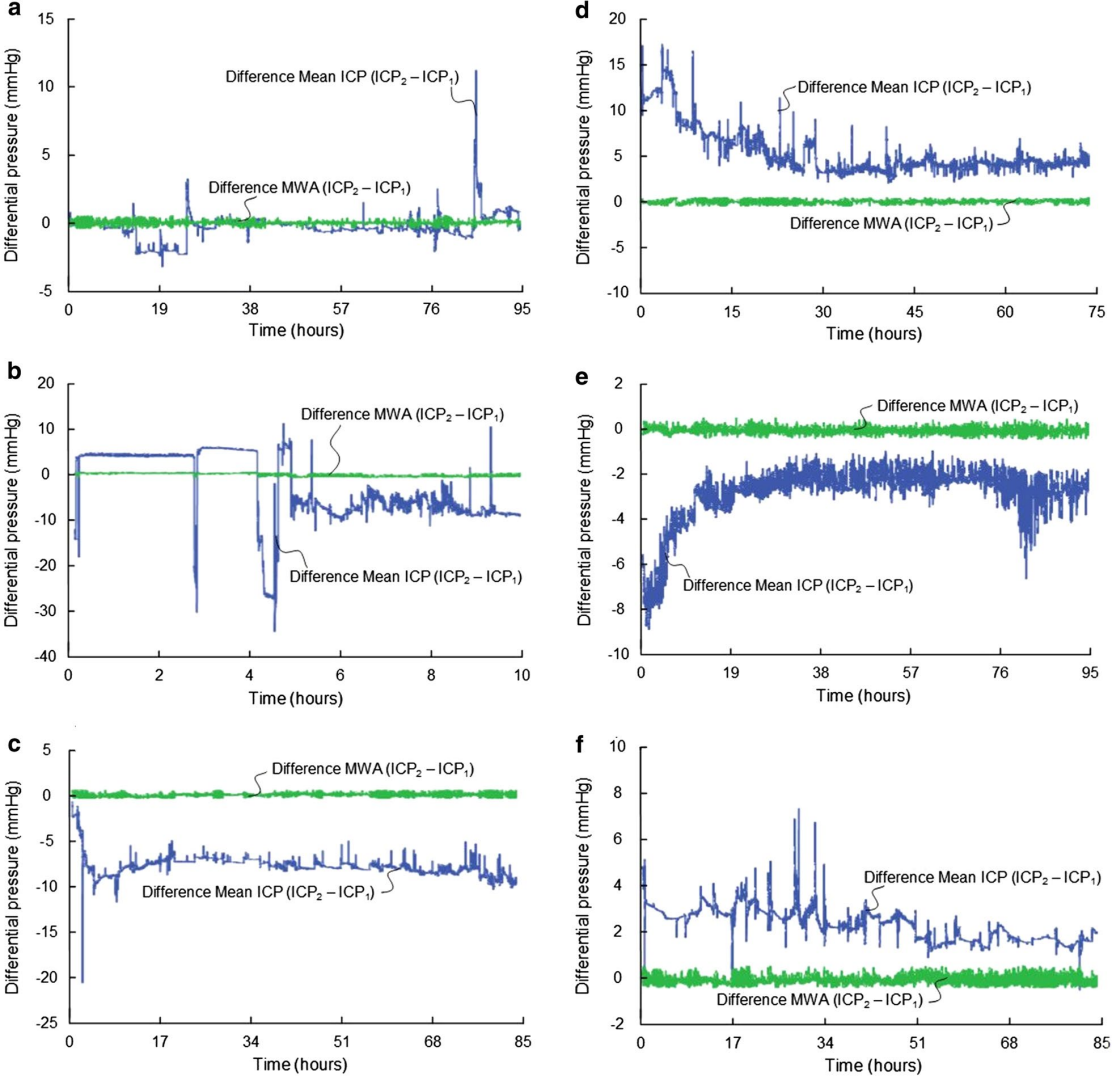
Occurrence of BPEs during ICP monitoring. In a prospective study, we examined the frequency and magnitude of BPEs in patients undergoing surveillance for SAH. Two Raumedic Neuro P sensors were placed nearby via the same burr hole in the skull. The different types of BPEs are illustrated. The trend plots in blue reveal differences in mean ICP computed for consecutive 6-second time windows (Mean ICP_Signal 2 –_ Mean ICP_Signal 1_), and the green plots show differences in MWA (MWA_Signal 2 –_ MWA_Signal 1_) of Signals 1 and 2, for the same 6-second time windows. The presence of PBEs is indicated by the differences in mean ICP, but with close to identical MWAs (differences in MWA < 0.5 mmHg). The red arrows indicate occurrence of BPEs. These plots are from different individuals. Type 2 BPE is shown in (**a**) and (**b**), while various examples of Type 3 BPEs are presented in (**c**), (**d**), (**e**) and (**f**). Adapted from Eide et al. [92]

To examine how frequent BPEs may be expected, we performed a prospective and observational study by inserting two Raumedic Neurovent P ICP sensors nearby in patients who underwent surveillance for aneurysmal SAH. We found that BPEs occur frequently in the clinical setting [92]; BPEs of a magnitude that might erroneously affect patient management was seen in nine of 16 patients (56%). Examples of BPEs from six of the 16 patients are shown in Fig. 7. The BPEs may explain the abrupt shifts in the relationship between mean ICP and MWA that occur when monitoring ICP with only one ICP sensor [93].

How can it be that changes in mean ICP are not accompanied with changes in MWA? The explanation is that these metrics are computed differently. While mean ICP is determined relative to a reference pressure, the MWA is an internal signal derived measure not impacted by the reference pressure. A sudden change in reference pressure only affects the zero level, not the ability of the sensor to read swift changes in pressures.

Given the potential risk that BPEs pose for patient management, it is surprising that this issue has hardly attracted any interest in the scientific literature. It is clear that BPEs causing significant alterations in zero pressure will impact the measured mean ICP scores.

The current ICP monitoring systems lack methodology for determining the occurrence of BPEs. Currently, it is therefore unclear how frequently BPEs occur. Our prospective cohort study [61, 92] suggests that it represents an underestimated problem that might significantly affect the clinical value of ICP monitoring. The reasons that BPEs have been given limited interest may be that single ICP wave analysis and identification have not been implemented in the current ICP monitoring systems. In addition, BPEs become easier to detect when measuring from two simultaneous ICP sensors, which is a rare occurrence.

### Baseline pressure errors and mean ICP derived scores

**Fig. 8:**
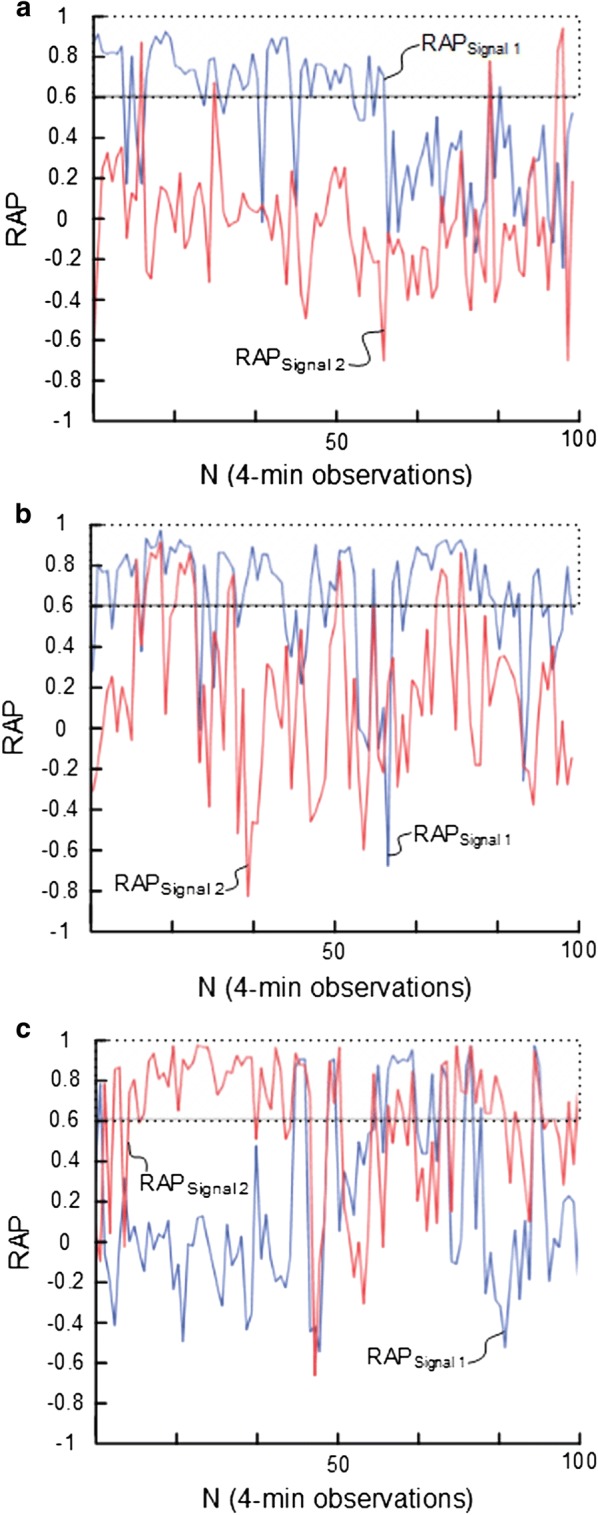
Impact of BPEs on determination of the mean ICP-derived score RAP. From different individuals undergoing ICP monitoring as part of surveillance of SAH, RAP was measured from two nearby Raumedic Neurovent P sensors placed via the same burr hole. Thereby the sensors measure ICP from the same compartment without pressure gradients. Trend plots of RAP [correlation coefficient (R) between the intracranial pressure (ICP) wave amplitude (A) and the mean ICP level (P)] of signals 1 and 2 are presented for three individuals. RAP was determined during 100 consecutive 4-minute periods for signals 1 (blue line) and 2 (red line). The horizontal lines at RAP 0.6 illustrate a commonly used upper normal threshold for RAP. **a** In this individual, the average of RAP_Signal 1_ was 0.50 (blue line) and the average of RAP_Signal 2_ − 0.04 (red line). **b** In this individual, the average of RAP_Signal 1_ was 0.64 (blue line) while average of RAP_Signal 2_ was 0.16 (red line). **c** In this individual, the average of RAP_Signal 1_ was 0.17 (blue line), and the average of RAP_Signal 2_ 0.59 (red line). Adapted from Eide et al. [61]

The BPEs not only impact mean ICP, but also all mean ICP-derived scores, such as PRx and RAP [76]. For example, the RAP index is heavily affected by BPEs [61]. Simultaneous ICP measurements from two separate ICP sensors placed nearby in the same patient showed marked differences in the calculated RAP values [94]. The differences were observed for all the ICP sensors that were tested (Codman ICP microsensor, Camino fiberoptic ICP sensor, Edward’s fluid sensor, and the Spiegelberg ICP sensor). Some authors speculated that RAP from different ICP sensors might reveal different values due to variation in pressure–volume reserve between different intracranial compartments [95]. However, our study [94], in which ICP sensors were also placed nearby, indicated that this explanation is unlikely. Nevertheless, a follow-up and prospective study including placement of two nearby ICP sensors (type Raumedic Neurovent P) within brain parenchyma for surveillance of individuals with aneurysmal SAH demonstrated that the RAP index computed from the two nearby ICP sensors differed (see Fig. 8). For example, in 50% of the patients, the combination RAP > 0.6 in ICP sensor 1 and RAP < 0.6 in ICP sensor 2 was observed in about one in ten RAP observations [61]. Moreover, for one in three individuals, the difference in RAP between ICP sensors 1 and 2 was ≥ 0.4 in 8% of RAP observations [61]. Individual examples of trend plots of RAP from three individuals are illustrated in Fig. 8. The differences in RAP are related to the fact that reference pressure variability affects the mean ICP. The authors concluded that these results make the RAP index less useful as a clinical parameter.

### Impact of pressure gradients on ICP measurements

Another important issue when evaluating ICP monitoring modalities is the possible role of pressure gradients on the ICP scores displayed to the health care personnel. In this regard, some questions arise: what is the value of measuring supra-tentorial ICP in subjects with infra-tentorial mass lesions, and how representative is an ICP measured in the right hemisphere in an individual with a lesion in the left?

A growing lesion will create pressure gradients needed to displace tissue and fluids, which in turn will affect the measured ICP. In this situation, the derived ICP scores will depend on the location of the ICP measurements. This concern is relevant both in TBI and in evolving hydrocephalus [96]. Consequently, rapid treatment should be considered in patients with growing localized lesions and accompanied with symptoms, despite any discrepancy between clinical symptoms and ICP.

Moreover, pressure gradients may exist between the cranio-spinal compartments and should be kept in mind when making important clinical decisions. Comparing pressure scores between the intracranial and lumbar compartments revealed differences in both static ICP and ICP wave amplitudes [97].

In the case of pressure gradients, there should be differentiation between static and pulsatile gradients in ICP. Hence, the static ICP is affected by hydrostatic pressure differences to a different extent than the pulsatile ICP. This question has been studied in the context of hydrocephalus, and particularly whether an outward pressure gradient can explain growing ventricles. In chronic cases, there were no trans mantle gradients in static ICP in individuals with communicating or non-communicating hydrocephalus [98], and no trans-mantle gradients in ICP wave amplitudes in communicating hydrocephalus [99]. Others reported that gradients in static pressure between the ventricular and parenchymal compartments could be attributed to hydrostatic pressure gradients, while differences in ICP wave amplitudes were minor [58]. Accordingly, the results may depend on the ICP scores in question.

### Pulsatile ICP

The term pulsatile ICP refers to the pressure changes occurring during the cardiac cycle. Each heartbeat results in intracranial pressure variations in accordance with the cardiac cycle measured as the ICP waveforms (see Fig. 3 and Additional file 1: Movie 1). Typically, a continuous ICP signal varies over time, characterized by a diastolic minimum pressure value and a systolic maximum pressure value, causing the calculated ICP scores to vary. This is further illustrated in Additional file 2: Movie 2. Established attributes from the single wave amplitudes are the amplitude (pressure difference between diastolic and systolic pressures), the rise time (time from diastolic to systolic pressures) and rise time coefficient (amplitude divided by rise time, providing a measure of the steepness of the ICP waveform) [32]. With regard to the morphology of the ICP wave, the most commonly studied ICP waveform attributes are the relative height of peaks P1, P2 and P3.

How the ICP waveforms are presented in the clinic varies according to the different monitors and devices. Some monitors show a few seconds of data providing poor time resolution, while others present long-time series with poor spatial resolution, which makes it a challenge to access the various morphological features. Some ICP equipment allows for storage of the ICP waveform data for post-processing, which is beneficial for research but has limited value in daily clinical practice.

**Fig. 9:**
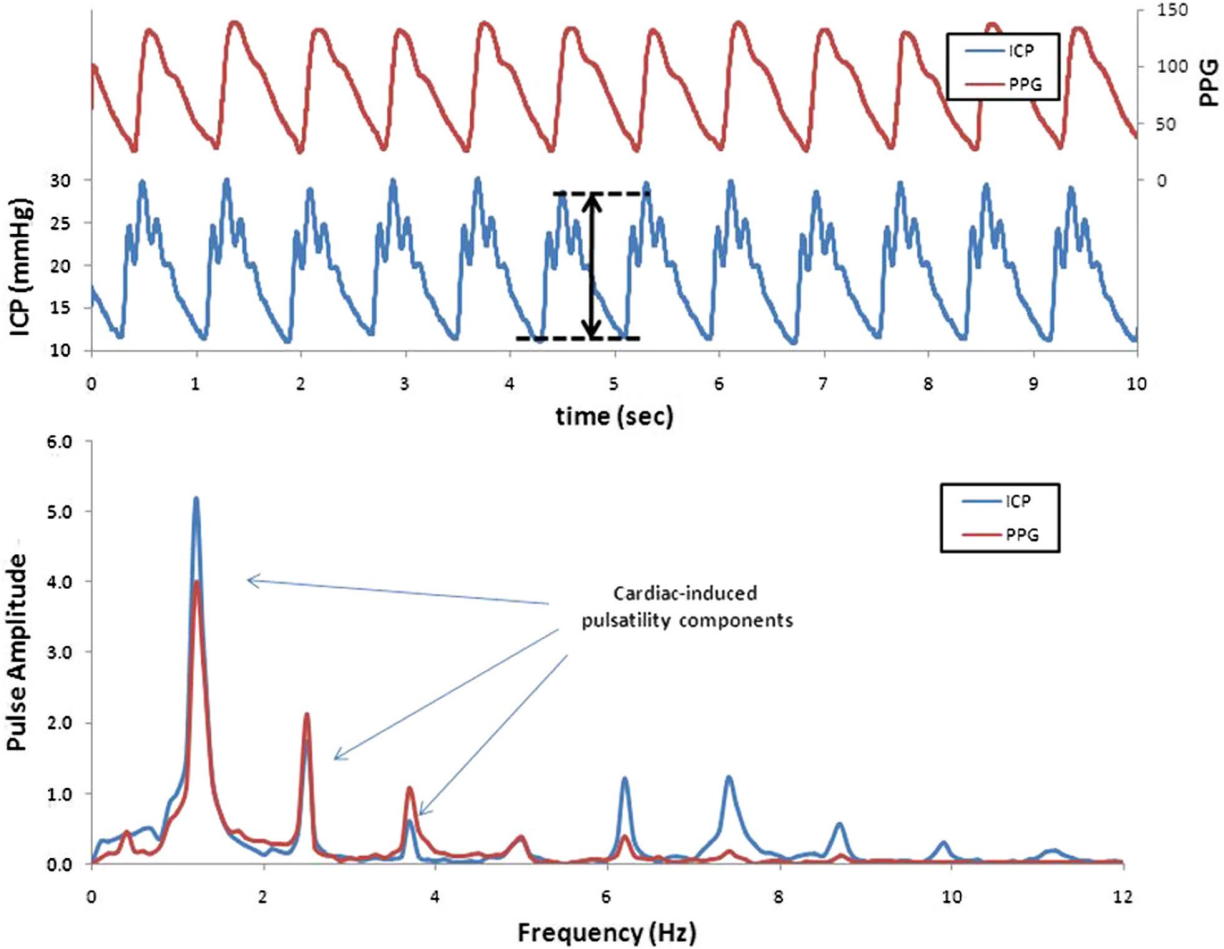
Differences between the time- and frequency-domain methods for estimating ICP scores. The pressure waveforms are usually presented in the time domain (upper panel). The single ICP waves are shown as the blue waveform and the arterial BP as the red waveform (PPG, photoplethysmograph, in this case). The lower plots present the pressure data analyzed in the frequency domain, represented as a function of frequency. The signal and the defined cardiac components separated from low frequency components such as respiration can be analyzed independently. Additional information available with frequency domain analysis is the phase, the frequency domain analog of timing in the time domain (not shown). The phase plot allows analysis of timing differences between the ICP and the reference waveform for each identified frequency component. From Wagshul et al. [32]

In the case of invasive ICP monitoring, the most studied ICP waveform parameter is the ICP wave amplitude. This has been defined and studied in different ways throughout the past decades, but the amplitude metrics AMP and MWA are the main references in the clinical literature [100]. The single wave amplitude (AMP) is determined in the frequency domain [63, 73]. The first harmonic of the frequency (Fourier) spectrum corresponds to the heartbeat, and the value of the first harmonic can be used to estimate the single wave amplitude. Mean ICP wave amplitude (MWA) is determined in the time domain, following the identification of the single ICP waves [101]. The average of identified single wave amplitudes over a defined period (6 s) is the MWA. These methods are not equivalent and provide different measures of the ICP wave amplitude [100], which constitutes an obstacle when comparing results in the literature. The clinical utility of ICP wave amplitude (AMP or MWA) has been implemented in only a few institutions. To date, the transition to clinical decision making has been made only in a few locations. Differences between the time and frequency domain methods are further illustrated in Fig. 9.

One RCT [102] provided evidence of improved outcomes after SAH when ICP-guided management was conducted according to MWA rather than mean ICP. Measurements of MWA are also used for selecting individuals with CSF problems for shunt surgery [25].

One additional algorithm is the computational algorithm referred to as Morphological Clustering and Analysis of ICP (MOCAICP) that assesses the morphology of the ICP waveform (e.g. ICP wave amplitude, slope, between peak time) and has been used in an attempt to forecast increased ICP [103].

The normal values of mean ICP wave amplitude (MWA) have not been determined since measurements cannot be performed in healthy individuals. The thresholds determined from our experience refer to MWA scores obtained in individuals who have undergone ICP monitoring, but where no evidence of abnormality was found. These individuals may therefore be considered “as normal as possible”. In general, we consider MWA values < 4 mmHg as normal with limited variation over age [24, 25, 67–69]. Notably, the relationship between MWA and mean ICP is U-shaped. When mean ICP becomes very negative, the mean ICP wave amplitude tends to rise [72]. The mechanisms causing ICP wave amplitudes to increase with very low mean ICP are not yet fully ascertained.

### ICP waveform derived scores

As for mean ICP, ICP waveform-derived indices have been introduced. The IAAC index is the moving correlation between ICP and arterial BP wave amplitudes (intracranial arterial amplitude correlation, IAAC) [104] and is assumed to provide information about the pressure–autoregulatory state. In patients with SAH, the outcome was impaired in individuals with elevated IAAC [105]. A comparable parameter based on AMP of ICP and ABP source signals denoted as PAx has also been introduced [106]. These indices have been studied to a lesser degree than mean ICP-derived indices such as PRx. One advantage of the ICP waveform derived indices, as compared to the ICP-derived indices, is the independence of reference pressure variability.

### Limitations with pulsatile ICP scores

The limitations related to the assessment of single ICP wave metrics involve the physiological processes creating single ICP waves and technological issues related to the proper identification of single ICP waves.

### Influence of physiological variables on single ICP waves

We have limited knowledge of the mechanisms affecting single ICP wave morphology. Since the early exploration of single ICP waves in the 1970s, experimental evidence suggests that the single ICP waves are affected by cerebral blood volume changes [107], and that cerebral blood volume therefore may affect the ICP wave amplitudes [108, 109]. The Cambridge-group reported that the ICP wave amplitude is more dependent on cerebral blood volume changes in individuals with TBI than in iNPH [108]. On the other hand, the ICP wave amplitudes in individuals with iNPH were not related to cardiovascular parameters such as arterial blood pressure, cardiac output, stroke volume, oxygen consumption and systemic vascular resistance [110].

Research indicates that the pulsatile ICP waveforms may also be affected by body position [72] and day-night cycles. As arterial BP waveform is the input signal to the ICP waveform, changes in vascular wall properties (vascular compliance) could also impact the ICP waveform. Given the influence of all physiological variables on the ICP waveform, it is not surprising that plotting MWA over time also reveals time-related variation (see Fig. 4).

As already commented on, the single ICP waves are affected by the intracranial compliance as well as the vascular compliance [see “Intracranial compliance (ICC)” section].

### Defining the single ICP waves

Today’s monitoring systems typically do not incorporate automatic methods for single ICP wave identification (or identification of corrupted waves caused by movement or other noise sources) or automatic procedures for assessment of reference pressure variability. Therefore, the question of the degree to which these aspects impact the utility of pulsatile ICP monitoring remains unanswered.

One limitation for monitoring of single ICP wave-derived parameters in the clinical context is a methodology for identification of the single ICP waves. Regardless of the method used to measure ICP (invasive, less invasive or non-invasive), the limited control of the ICP source signal means that the ways by which to control the information provided to the health care personnel are limited. The ICP source signal may be corrupted for a number of reasons. Clearly, erroneous ICP scores provided to the health care personnel may result in inappropriate patient management.

One main avenue for improvement of ICP monitoring practice is through incorporation of automatic algorithms for single wave identification along with ICP waveform analysis becoming part of standard practice, which would enhance control of the ICP source signal. At the Department of Neurosurgery, Oslo University Hospital, automatic identification of single ICP waves was introduced in 2005 [101]. The present review is largely based on the clinical experience done in this department throughout the early 2000s. Automatic identification and characterization of single ICP waves represents a challenge that requires a dedicated methodology [101]. For example, determining pressure differences from the bottom and top of non-identified waves may cause erroneous information because “waves” without an identification procedure may be noise waves and may not be related to physiological pressure waves. Such errors will affect the measurement of all wave attributes, including amplitudes, rise time and rise time coefficient. Since measurement of pulsatile ICP metrics is highly technology-driven, differences in methodologies represent a limitation in terms of the build-up of knowledge.

However, depending on how ICP wave amplitudes are determined, these scores do not appear to be influenced by zero pressure levels [45, 90]. When single wave amplitudes are determined as relative values within the pressure signal itself, the scores are not influenced by the absolute reference pressure.

### Slow waves

In the literature, the term “ICP waves” may refer to different ICP characteristics. While pulsatile ICP refers to the pressure fluctuations during the cardiac beat contradiction, respiratory waves are low frequency fluctuations in static mean ICP related to respiration. Lundberg also differentiated between A, B and C waves, which are slow and semi-periodic alterations in mean ICP, not referring to pulsatile ICP.

### The respiratory wave

Respiratory waves occur at a slower frequency than the cardiac part of the ICP signal with a frequency of about 0.3 Hz (provided there are 20 respiratory cycles/minute). In the context of ICP monitoring, these respiratory waves are referred to as slow waves [63]. Assessment of these waves has not been implemented in current monitoring systems because they require dedicated software. Therefore, the assessment of respiratory (slow) waves has come into clinical practice only to a very limited extent and is primarily done for research purposes.

### B waves

**Fig. 10:**
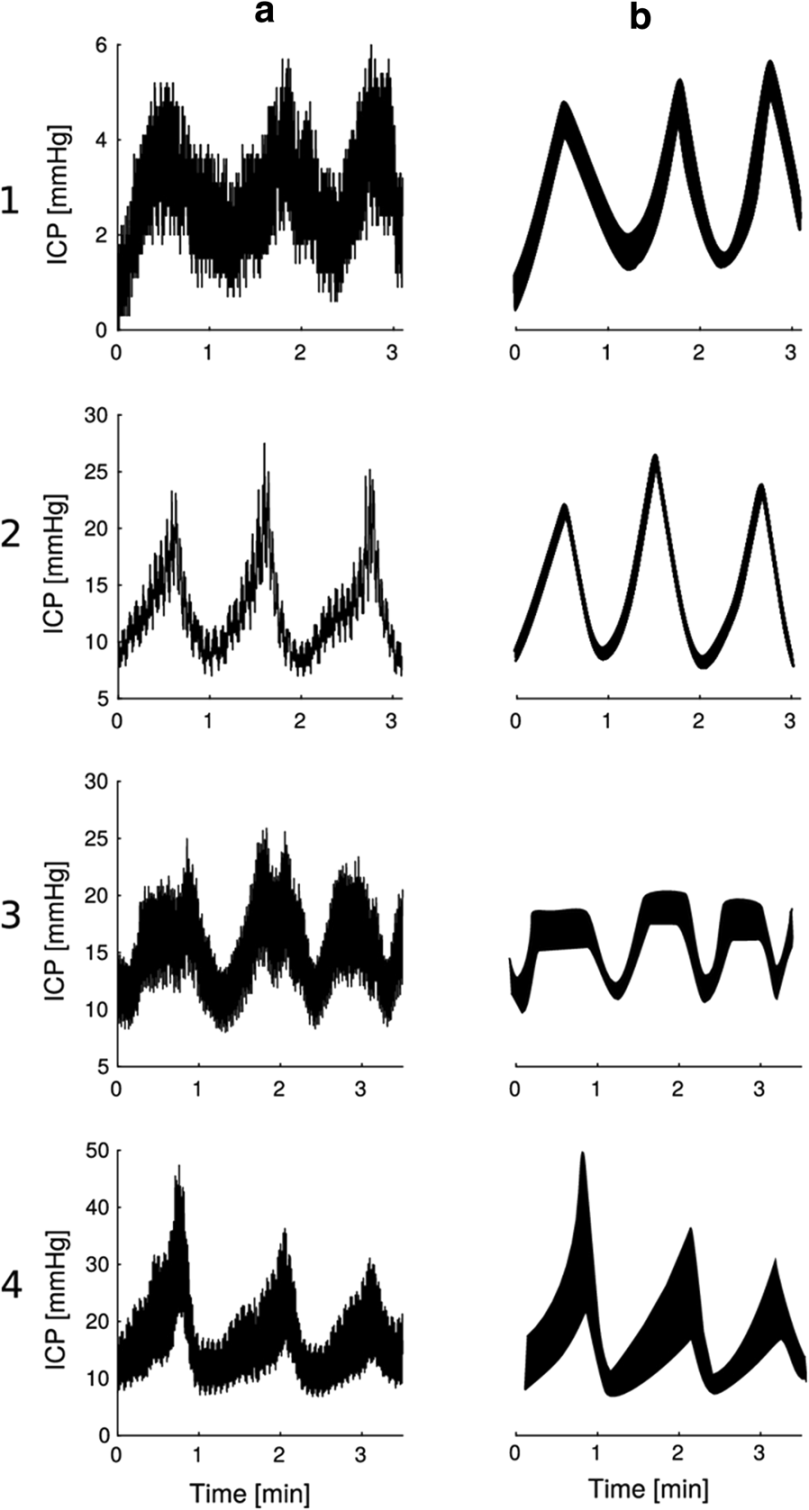
B waves are elevations of mean ICP that may have different patterns. Column A illustrates the trend of mean ICP and column B the computer-generated examples. (**1**) B waves with symmetrical shape and amplitude < 10 mmHg. (**2**) B waves with symmetrical shape and amplitude > 10 mmHg. (**3**) Symmetrical B waves with plateau. (**4**) Asymmetrical B waves. The timescale is in the order of minutes. It should be noted that amplitudes of B waves and single ICP waves are fundamentally different. From Martinez-Tejada et al. [112]

Lundberg differentiated between three types of static ICP elevations, namely A, B and C waves [13]. The A waves are denoted as plateau waves or vasogenic waves occurring during very high ICP (> 50 mmHg), the B waves are short-duration elevations in ICP (0.5–2 waves per minute) with variable pressure levels up to 30–50 mmHg. C waves are more frequent (about 4–8 waves per minute) elevations of mean ICP (up to about 30 mmHg). While attention has been given to the role of B waves [111], it has been difficult to incorporate assessment of B waves clinically because of difficulties in defining and quantifying them. A recent review addressed the diversity in definitions of B waves and the variability of their presentation [112]. B waves may have different patterns (Fig. 10), which creates problems in identifying and characterizing them. Different notations have been used to describe B waves such as “slow waves,” “vasogenic waves,” “ICP waves” and “B slow waves” [112]. B waves should therefore not be confused with single ICP waves (or pulsatile ICP). The clinical implications of the B waves frequency and magnitude content is a research question that remains to be determined.

### Intracranial compliance (ICC)

There are currently limited options for measuring ICC directly in the clinical context, although this has been an objective since the introduction of clinical ICP monitoring in the 1960s. It has proven to be difficult to measure, however, without causing too much damage. The different approaches to measuring ICC are briefly mentioned in the following sections.

The experiments required to investigate the intracranial pressure–volume relationship were highly invasive and was first explored in animal experiments [113–117]. The animals were exposed to an intracranial volume increase with simultaneous measurement of ICP, which demonstrated a non-linear pressure–volume relationship (see Fig. 1). Based on the experimental studies on intracranial volume–pressure relationships, the volume–pressure test (VPT) described the increase in ICP caused by administration of a minor volume of fluid to the ventricular CSF. From this, less invasive clinical approaches to assess ICC evolved. Different scores for the pressure–volume reserve in the clinical situation were developed, including the pressure–volume index (PVI) [115], and volume–pressure response (VPR) wherein 1 ml was added or subtracted from the ventricular CSF [118]. From the studies utilizing minor intracranial volume changes for ICC assessment [119], a commercial product for ICC measurements (Spiegelberg Brain Compliance Monitor; Spiegelberg GmnH, Hamburg, Germany) was introduced to the market. This product incorporated a technology for inflating/deflating a balloon connected to an ICP sensor/drain in the CSF [120]. Favorable results from the clinical use of this monitor were reported [121]. However, a major drawback with these methods of assessing ICC was the need to add or subtract volume in the intracranial compartment, which is invasive and risk-related. In some clinical situations, even a minor volume change in individuals with impaired ICC might cause a harmful increase in ICP.

Another approach to obtain information about ICC while avoiding artificial intracranial volume changes is deciphering ICC from the single ICP waveform characteristics [122, 123]. Over the years, different approaches were reported: Szewczykowski et al. [124] presented a computerized method enabling analysis of the peak–peak pulse amplitude as a function of mean ICP wherein the slope of the amplitude pressure curve was considered indicative of the ICC. According to this concept, the intracranial volume (dV) change per heartbeat was assumed to be rather constant, and the peak-to-peak amplitude an indicator of the ICC.

Later, Czosnyka et al. [125] introduced the RAP index, which is the moving correlation between pulse amplitude and mean ICP over 4-minute time periods. An index approaching 0 is thought to be indicative of good ICC, while an index approaching +1 is considered indicative of impaired ICC [63]. The calculation of RAP was determined from the frequency domain method and involves extracting the amplitude of the fundamental frequency. Another method based on the frequency domain method is determining the centroid of the ICP power spectrum (between 4 and 15 Hz), denoting the high-frequency centroid and introducing it as an indicator of the ICC [126]. Mortality after TBI increased with an increasing mean high-frequency centroid value [126].

Cardoso et al. [127] used an approach based on separation of the typical peaks P1–P3 of the single ICP waveform. According to this concept, when ICC is impaired, the tidal (P2) and dicrotic (P3) peaks exceed the systolic peak (P1) with the disappearance of the dicrotic notch, while the systolic peak (P1) exceeds the tidal (P2) and dicrotic (P3) peaks under normal conditions. Others have more recently performed automatic identification of the ICP waveform peaks using an artificial neural network [128], and confirmed an association between peak separation and ICE, but no relationship with resistance to CSF outflow.

Given the many approaches to quantify ICC [63, 123, 126, 129], evaluation of the ICP wave amplitude is one approach that has made its way into clinical practice [60, 105]. In our institution, we have used the mean ICP wave amplitude (MWA) as a proxy of ICC. According to this concept, the MWA represents the pressure response to a net intracranial blood volume change of about 1 ml during each cardiac beat. The MWA was found to correlate with the ICC measured by the Spiegelberg compliance monitor [130], and with intracranial compliance computed during ventricular infusion testing [131]. Since all physiological parameters vary over time, including the net intracranial blood volume change, we have incorporated monitoring of MWA over many hours, usually overnight, when monitoring is done for diagnostic purposes. The MWA is computed every 6 seconds, multi-hour monitoring, which provides several thousands of observations that may reduce the impact of variation over individual cardiac cycles.

When comparing three metrics of ICC (i.e. ICP wave amplitude, RAP and ICP slope) related to outcome after TBI, the ICP wave amplitude was found to have the best performance [132]. As similar ICP levels can correspond to different ICC states, ICC monitoring could provide significant improvement in patient care as it would allow for early intervention in progressively worsening patient states [133].

Another research line has been to infer ICC from phase-contrast magnetic resonance imaging (MRI). Based on fluid flow rate estimations in CSF and blood going to/from the brain, estimated pressure and volume changes have been used to estimate ICC [134]. However, the non-invasive estimation of ICP wave amplitudes by phase-contrast MRI was not found feasible by others [135].

Further studies are needed to determine the clinical benefits of ICC monitoring. In our institution, we have found measuring ICP wave amplitudes, indicative of ICC, clinically useful for prediction of shunt response in iNPH [25]. Moreover, a randomized controlled trial showed significantly improved outcome in individuals with SAH who were managed according to MWA, as compared to traditional mean ICP guided management [102].

## Less invasive ICP source signals

The need for surgical penetration of the skull and dura for placement of parenchymal or ventricular sensors for extended periods of time limit the application of ICP measurements, especially outside of the ICU, where the bar for neurosurgical intervention is high. This raises the need for alternative approaches to accessing information about ICP.

### Lumbar puncture

The most widespread use of measuring ICP is via lumbar puncture (LP) and involves advancing a needle into the lumbar intrathecal space, which is linked on the other end to an external pressure transducer. The common way is to measure fluid level as centimeters of water (H_2_O) and use this as an indication of ICP. A requirement for this measurement methodology to provide relevant results is obstruction-free CSF communication pathways. Estimating ICP from LP is common in neurological practice, and is widely used for assessing ICP in individuals with idiopathic intracranial hypertension (IIH) events, although the limitations involved in estimating ICP by LP are well known [136].

Lumbar CSF pressure measurements during so-called infusion tests also have a long tradition [137]. In such procedures, the CSF pressure is measured during infusion of a fluid to the lumbar compartment and the pressure change in response to the administered fluid is interpreted as resistance to outflow of CSF. This is performed on a routine basis in several centers [63]. The main indication is to assess shunt dependency or shunt failure in individuals with tentative CSF circulation failure.

The literature is somewhat divergent as to how well lumbar CSF pressure scores compare with ICP scores. The pressure measured by LP depends on the position of the lumbar region relative to the head since hydrostatic pressure differences will determine how lumbar CSF pressure compares with ICP. It has been reported that CSF pressure measured by LP compares very well with ICP [27], while a similar perfect match was not reported by others [97].

### Limitations

Serious complications are rare, but LP is contraindicated in cases when very high ICP is suspected due to the possibility of brain herniation [138]. If the CSF pathways are obstructed, ICP will not be measured correctly with this procedure. Further, this is not strictly an approach for ICP measurement as it is performed in the spinal region.

The most important question is the extent to which LP measurements may correctly estimate ICP. LP measurements have been found to vary with mean ICP [139] and the mean ICP wave amplitudes differed from the mean CSF wave amplitudes measured in the lumbar compartment [97]. LP measurements with current devices, however, only reflect an instantaneous ICP value and are therefore not useful for continuous mean ICP monitoring in its current state.

### Epidural ICP monitoring

Placement of an ICP sensor outside the dura mater is less invasive than placing a sensor within the brain parenchyma or ventricular CSF. As sensor placement in the epidural space would reduce the risks of subdural or parenchymal hemorrhage, epidural placement was explored in the earlier years of ICP monitoring. Patients with increased risks of internal bleeds such as those with acute liver failure [140] or hemorrhagic diseases could theoretically benefit even more from such a sensor placement.

However, since the introduction of epidural ICP sensors, numerous studies have reported errors in mean ICP monitoring [141, 142], typically reporting ICP as too high. Epidural ICP monitoring was therefore discontinued in most centers, although some centers have demonstrated that epidural ICP measurements provide very accurate readings with regard to pulsatile ICP, both with parenchymal probes placed epidurally [143] and with commercially available epidural probes [144]. This could be beneficial for certain patients and should therefore not be disregarded completely.

### Limitations

Epidural ICP measurements also require a trepanation. While the risk of bleeds is reduced, it is not eliminated. There is also a corresponding risk of infection.

Epidural ICP monitoring highlights the importance of ICP source signal control because the handling of the source signal determines the safety of the information provided. The major problem with epidural ICP sensors relates to the ability to offer reliable mean ICP measurements [143, 144]. This is because of issues related to zero reference pressure. However, measurements of epidural ICP wave amplitudes are feasible and accurate but require dedicated software because an algorithm for single ICP wave identification is needed [143, 144]. Accordingly, even though it is considered that epidural ICP measurements do not provide valid mean ICP scores, experience from measuring ICP wave amplitudes has illustrated the value of the technique. The results are better when using dedicated epidural ICP sensors rather than ICP sensors designed for parenchymal use [143]. The limitations in using epidural ICP measurements are therefore related to static ICP measurements, not to measurements of the ICP waveform. Epidural ICP measurements are increasingly used in preclinical models utilizing ICP monitoring.

## Measurements of ICP in animals

Preclinical long-term measurements of ICP in animals is required to understand the normal regulation of ICP, as well as mechanisms behind abnormal ICP in brain disease or injury. Animal models have utilized fluid-filled ICP catheters placed in the ventricles, cisterna magna, thecal sac, epidural and subdural spaces, and fiberoptic ICP measurement systems in brain parenchyma [145]. One study compared simultaneous measurements from ventricular, cisterna magna and parenchymal pressure measurement devices, and found the ventricular ICP monitoring preferable in terms of accuracy and least brain damage [146]. Another study reporting a novel method for epidural measurements of ICP in rats reported a strong correlation between ventricular and epidural ICP score [145]. More recently, telemetric devices utilizing subdural ICP catheters have been introduced for long-term monitoring of ICP in rats [147–149].

To obtain reliable data from preclinical studies of ICP, adherence to the methodological limitations are of the utmost importance. Similar methodological issues as seen in humans such as shifts and drifts in static ICP occur in animals. Preferably, preclinical studies should sample the continuous raw ICP signals and include assessment of both the static and pulsatile ICP after single wave assessment so as to ensure correct pressure assessments. However, in freely moving animals, high signal-to-noise ratio is expected.

## Non-invasive ICP source signals

When considering the limitations of current invasive ICP measurements, the implications for non-invasive ICP (nICP) monitoring should be included. Because of the risks related to invasive ICP measurements, numerous non-invasive ICP source signals have been explored. The potential benefits of non-invasive ICP monitoring seem clear. This section, therefore, discusses state of the art, the limitations and weaknesses of the nICP source signals, the nICP source signal control, and the issue of measuring absolute ICP non-invasively from a clinical perspective.

### Modeling approaches

As non-invasive approaches to ICP monitoring require other input data than direct ICP measurements, they all rely on a form of model that permits the non-invasively obtained source signals to be altered into signals or information that can be utilized by the clinicians. The first model of relevance was the three-compartment Monro-Kellie doctrine, which was a concise description of a highly intricate system. In the decades that followed, numerous models of varying complexity have been proposed. Some describe the cerebrovascular system using mechanistic models [150–154], while others rely on statistics and ideally huge amounts of data in order to identify top-level statistical relations the clinicians can utilize [155–157]. Both categories are included when various approaches to non-invasive ICP estimation are described in the following sections. While model-based estimation clearly has the advantage of resonating with clinicians and aiding everyone in understanding the mechanics of the intracranial compartment, the black box models are an exciting approach as digitalization of health data and immense computational power is becoming a reality.

### Arterial blood pressure waveforms as nICP source signals

Several studies have explored the use of various arterial BP waveforms as nICP source signals. This approach seems logical, given that the arterial BP waveform serves as an input signal to the ICP waveform. Mathematical approaches have been explored, including the use of central aortic BP waveforms [158]. However, we did not find that central aortic BP waveforms could be used as an nICP source signal [157] and the use of arterial BP measures alone seems less useful. We also stress the role of assessing and comparing the pressure waveforms and their time alignment when conducting such and similar research [159].

### Transcranial Doppler and cerebral blood flow velocity

The use of arterial BP signals has been combined with other non-invasive signals, in particular, transcranial Doppler (TCD). The TCD technology was initially developed as a non-invasive tool for vasospasm detection after SAH and for evaluating cerebral circulation [160]. It utilizes the principle of the Doppler effect. Cerebral blood flow velocity (CBFV) is measured by using the changes in frequency that occur due to the blood’s movement. As ICP can affect the blood flow and the cross-section of vessels, CBFV can provide added information about the state of the intracranial space. There have been several approaches to nICP estimation using both CBFV waveform characteristics and CBFV [161–163]. The TCD pulsatility index (PI) was not found useful to estimate ICP. This is related to the fact that the physiological parameters (vessel compliance, autoregulation, and arterial BP) vary over time [164]. A recent study, however, reported that a combination is superior to using only one of the metrics [165]. An approach combining radial artery BP and CBFV waveforms with a mechanistic model has also provided promising results [166, 167] with regard to mean ICP estimation.

Another approach for estimating nICP using Doppler technology is the two-depth ophthalmic artery Doppler ultrasonography developed by Ragauskas [168]. A more recent study validating this technique reported a good correlation between invasive and nICP scores [169]. However, pulsatile ICP measurements are not possible with this approach, and continuous monitoring of CBFV waveforms must be improved before the technique can be clinically useful. The use of TCD is also not possible in certain sub-populations [170] because a cranial window is required.

### Limitations

It is well known that TCD is highly user-dependent as results depend on the direction by which vessels are approached. Even minor changes in the direction of the probe may significantly affect the measured Doppler signal. Moreover, CBFV is significantly affected by other changes in physiology such as medications, autoregulation, and hyperemia. Therefore, the use of TCD as a source signal for nICP is associated with many unresolved challenges. Therefore, it seems infeasible to monitor nICP over several hours.

### Otic methods

The cochlear aqueduct represents a connection between the CSF in the intracranial cavity and the inner ear, which allows for CSF exchange when patent. Utilizing this pathway for nICP estimation was first attempted by Marchbanks, who looked at the tympanic membranes’ response to excitation of the stapedial reflex and how this varied with ICP level [171]. This technique has since been used as a tool for mean ICP assessment in various studies with variable, but not convincing, clinical results [172–174].

**Fig. 11:**
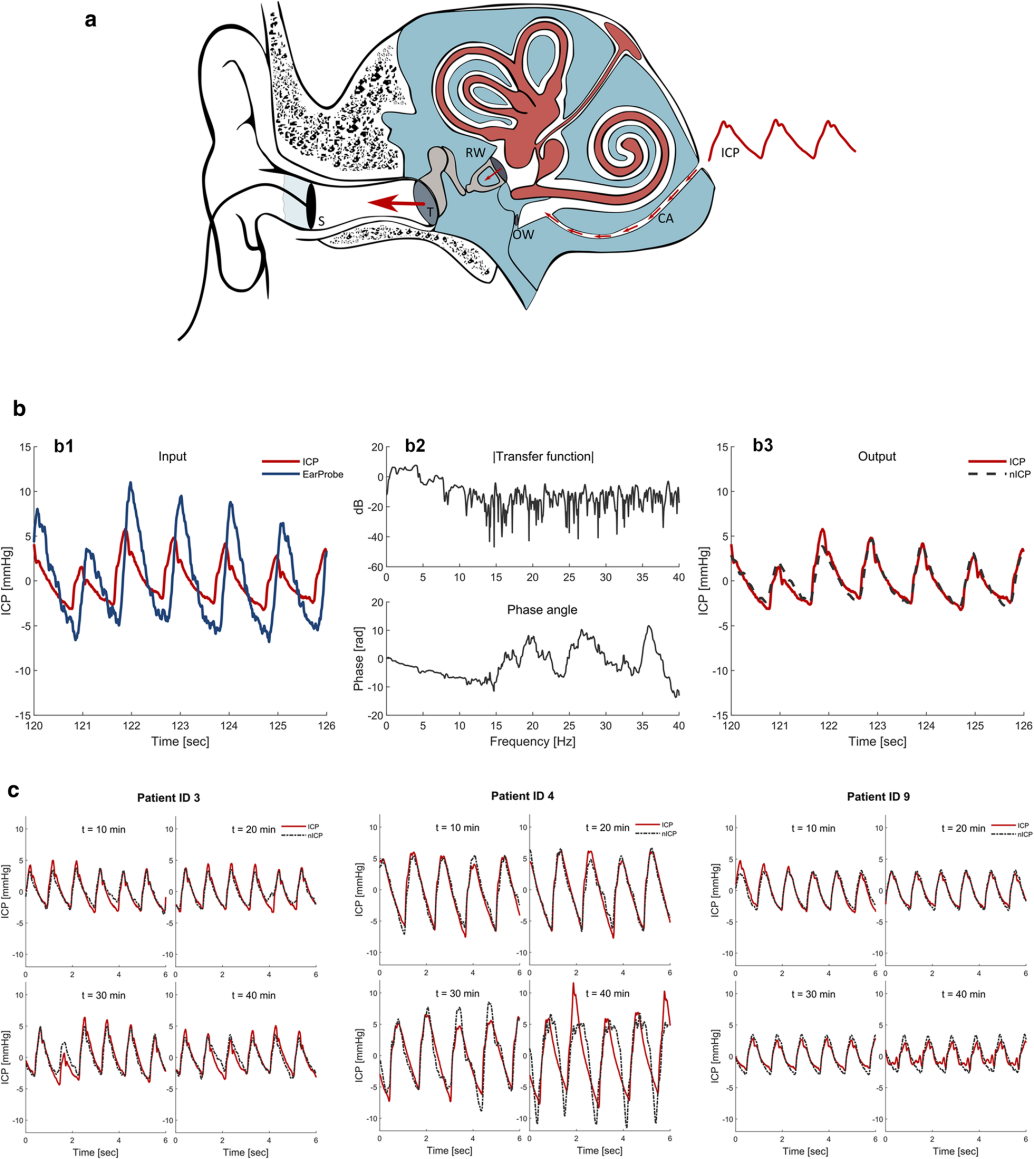
Tympanic membrane pressure (TMP) as a surrogate marker of intracranial pressure. (**a**) Schematic illustration of the anatomical structures involved in measurements of TMP waveforms. The non-invasive TMP waveforms were measured in the outer ear and used as input for the estimation of non-invasive ICP. *ICP* ICP input signal, *CA* cochlear aqueduct, *OW* oval window, *RW* round window, *T* tympanic membrane, and *S* sensor. (**b**) An example showing 6 seconds of the input signals and the corresponding transfer function estimate based on a total 10 minutes are shown in b1 and b2, respectively. The resulting output from the combination of b1 and the inverse of b2 is presented in b3. (**c**) The non-invasive ICP waveform estimate (nICP, interrupted red line) is shown together with the invasive ICP waveform (continuous red line) for four different 6-second time windows after the beginning of the measurement. The time delay between the nICP and ICP signals, as seen in b1, has been removed for visual comparison. Adapted from Evensen et al. [177]

Some approaches to non-invasive estimation of ICC through the otic connection have also been presented in the literature. Davids et al. reported that pulse waves in the outer ear that were possible to measure changed shape when the patient was tilted (and thereby, their ICP was changed) [175]. Direct assessment and comparison of ICP waveforms and tympanic membrane pressure waveforms have also been conducted [176, 177] and found to have limited clinical potential due to the patient dependent cochlear aqueduct. In our study (Fig. 11), we compared tympanic membrane pressure waveforms with the invasive ICP waveforms and found a low degree of similarity [177].

A similar technique to the stapedial reflex base proposed by Marchbanks aims to use the change in otoacoustic emission that occurs when ICP changes. The principle behind this technique is that evoked CSF pressure in the inner ear will alter the load on the stapes and, as a result, the sounds generated in the inner ear in response to sound excitation will change when ICP changes [178, 179]. However, this approach to nICP estimation is also, to a large degree, subject to significant interpatient variability.

### Limitations

Using otic source signals for nICP is dependent on a patent cochlear aqueduct, which serves as a mechanical filter for the transmission of ICP-derived signals. In most patients, this filter provides a substantial distortion of ICP signals. At present, the use of otic source signals for nICP estimation does not seem useful for clinical application.

### Imaging-based methods

Various radiological approaches have been proposed to identify increased ICP. Traditional measures such as ventricular size, however, are not useful. For example, in a study of 184 patients, no significant correlation was found between invasively measured ICP and the size of the cerebral ventricles measured by computed tomography (CT) [180], which is often highlighted as a symptom of elevated ICP. A study of 20 TBI patients by Pappu et al. [181] approached ICP estimation from a slightly new angle as they aimed to find features in CT scans associated with low ICP and thereby identify patients that could be excluded from invasive procedures. The study was, to some extent, able to differentiate between high and low ICP by assessing the relative CSF volume with a predictive accuracy of 67% for ICP < 20 mmHg [181].

A review of various radiological measures used to assess raised ICP in children was published recently [182]. One method that has attracted interest is combining MRI images with fluid mechanics to use the measures of blood and CSF volumes that enter and leave the brain during the cardiac cycle to compute a brain elastance metric that allowed for differentiating between elevated and normal ICP [134, 183]. However, contradictory results have been reported in other studies examining pulsatile ICP information from MRI images [135, 184].

Although potentially useful as a screening tool for very high ICP, brain imaging techniques alone are currently not reliable for clinical management [185, 186]. They are also not appropriate for continuous assessments.

### Limitations

Currently, ICP cannot be inferred from brain imaging modalities. If this became feasible, a significant flaw would be that it would only provide short-term assessments as repetitive measurements would not be possible. In addition, imaging modalities are costly, and thus not available in many settings.

### Acoustic methods

Acoustic-based approaches aim to estimate ICP from the acoustical properties of the skull or the constituents of the intracranial compartment. One approach, proposed by Levinsky et al. is a combination of acoustic and otic methods called transcranial acoustic signals (TCA) [187]. This method is based on a source signal of 621 Hz being sent from an earplug in one ear and received by an earplug in the other ear. The receiving earplug also recorded *head generated sounds*, which is important in the estimation of ICP. A training set, where both invasive ICP measurements and TCA were accessible, was used to establish a mathematical model. The non-invasive estimates of ICP were found after splitting the recorded signal into different frequency bands corresponding to different processes in the body (blood flow, breathing and test signal). The study found a mean difference of 0.39 mmHg and 0.53 mmHg for static ICP and pulsatile ICP, respectively, between estimated values and simultaneously recorded parenchymal ICP. However, further validation of the approach has not been presented.

Another well-documented approach is methods based on *ultrasonic time of flight* (TOF) measurements. The rationale behind these techniques is the assumption that the acoustic properties of the intracranial structures, as well as the size of the cranial vault, can change in the case of elevated ICP. Such changes should then affect the propagation speed and frequency attenuation of emitted ultrasound pulses [188–191]. This has produced good results in small scale studies [191]. One drawback of this technique involves variable data quality as well as its ability to only measure ICP relative to a known reference ICP value [59].

### Limitations

Currently, there are no validated acoustic methods for nICP measurement. The extent to which acoustic signals will become useful as source signals is a matter for future studies.

### Optic nerve sheath diameter (ONSD)

Another approach for non-invasive ICP estimation is the utilization of the window of the patient’s eye. The CSF of the intracranial cavity also surrounds the optic nerve, which in turn is surrounded by a sheath of meningeal layers. It has been demonstrated that when ICP increases, the radial pressure increases in the CSF surrounding the optic nerve, causing the diameter of the sheet to expand.

There have been several approaches to quantify this diameter using different imaging techniques such as MRI, CT, ultrasound imaging and optical coherence tomography. This approach has proven quite successful in separating the low and high levels of ICP by comparing population-averaged values to patient-specific measurements of the sheath diameter [192].

In addition to only measuring the diameter, pulsatile information has successfully been included in an aim to access information about the sheet’s stiffness [193, 194]. In particular, the ultrasound-based variant of this technique can be seen to have promising clinical value due to its applicability and accessibility. In the case of TBI, people who are not ICU specialists can perform these measurements within minutes.

One advantage of this technique, especially the ultrasound-based solution, is its applicability and accessibility. These measurements can be completed within minutes of a TBI and are possible without much medical training. A recent systematic review and meta-analysis [195] concluded that this technology is promising for nICP measurements.

### Limitations

The main drawback of this technique is that it is unfit for continuous measurements. In addition, its ability to separate between high and low ICP primarily makes it a triage tool and less valuable at the bedside of patients with less imminent diseases of the CNS.

It should be noted that when there is interocular ONSD asymmetry in the same subjects [196], it should be taken into account. Ideally, both eyes should be assessed, but this may be difficult in traumatic cases because facial and eye damage is often involved.

### Other approaches for nICP measurements

The nICP approaches referred to in this paper cover a subset of the contributions with the aim of highlighting the most promising and most investigated techniques. For example, a possible technique that shows some promise is near infra-red spectroscopy (NIRS) [197], which is a non-invasive technique for monitoring cerebral oxygenation, and potentially a source signal for nICP. A more comprehensive list can be found in other review papers [59, 186]. However, despite considerable effort over many years, these and other review papers [40, 174, 198–202] came to the same conclusion reached in this paper: no nICP technique manifests as a complete universal solution applicable for all patients in all situations. None of the current nICP methods is capable of providing real-time, calibration-free, long-term continuous ICP measurements, and especially not ICC measurements.

However, there are a few techniques that seem promising for specific clinical situations. For example, ICP evaluation appears feasible in triage situations where conventional ICP monitoring is unavailable. While trained physicians know how to recognize and manage patients with very high ICP, this might not be the case for individuals such as a soccer coach or for people operating in first-line health care. In cases of acute brain injury, the combination of portable ultrasound tools and measurements of ONSD seems promising [195] as it may allow people who are not medical professionals to distinguish high and low ICP. However, this approach is not suitable for continuous monitoring and does not provide any ICC metric.

One technique that allows for continuous ICP monitoring to a greater extent is the mechanistic model-based approach based on CBFV waveforms [166, 167]. However, long-term, reliable continuous monitoring of these is challenging with the current measurement equipment. In addition, this approach does not allow for any computation of an ICC metric.

There are a limited number of windows into the cranial cavity, especially in adults, that could allow for non-invasive monitoring of ICP. In this review, we therefore wish to highlight the field’s need to shift focus.

It is important to keep in mind that there are several different clinical areas for nICP monitoring, which has implications for how we assess the nICP source signals. In a pre-hospital setting or a hospital admittance department, the desire might be to determine whether abnormal ICP is present. This may be useful for triage and the need to determine further treatment. Evidence of very high ICP in wounded soldiers on the battlefield may be useful. However, the nICP equipment may not be very accurate in these situations. For example, the goal might be to determine whether ICP is above a certain level (e.g. 30–50 mmHg). For this particular purpose, the technique must be easy to operate, user-independent, practically risk-free, and independent of the operative environment. Conversely, long-term surveillance of abnormal ICP within the ICU over many days would require accurate measurements of ICP. For a new method to be adopted in neuro-intensive care, it must provide accuracy at a comparable level to the invasive gold standard and allow for continuous assessments of ICP, both in the ICU and at the bedside. Finally, assessment of individuals with chronic complaints (e.g. symptoms related to CSF problems) might be useful but would require accurate measurement results to be clinically attractive.

The source signals for non-invasive monitoring of ICP used to date have shown limited success. We conclude that the currently available source signals are of limited value.

## Invasive ICP source signals from implantable pressure sensors

Given that the comprehensive research on non-invasive ICP monitoring has not yet provided any technique that can be readily adopted in clinical practice, it is possible that current research should be shifted to smaller and more achievable goals. Minimally invasive techniques such as extradural measurements provide pulsatile ICP readings similar to parenchymal measurements (Fig. 2). Therefore, sensor development with this in mind appears to be a technological advancement that can be incorporated more easily into clinical practice than completely non-invasive mean ICP estimation. Sensor placement in the lumbar region rather than in the parenchyma will likely cause less immunologic response and tissue damage.

The issues related to ICP source signal control and reference pressure variability are even more important when it comes to implantable ICP sensors.

### Telemetric sensors and miniature sensors

For several neurological and neurosurgical diseases, improved patient care would include long term monitoring of ICP and ICC on timescales of weeks to months. The invasive catheter systems can only be used in the hospital setting. Measurements outside the hospital require implantable systems. This would allow optimal planning of a patient’s rehabilitation post-surgery, as well as early detection of any progressive worsening of the patient state, which can occur, for example, in hydrocephalus patients. While the risks associated with parenchymal and ventricular pressure monitoring are often tolerated before and during neurosurgical intervention because of the severity of the situation, these measurement modalities are unsuitable for measurements exceeding days/weeks due to the increased risks of infections accompanying the prolonged measurement duration. Telemetric devices have therefore been proposed as an alternative to the traditional invasive cable bound techniques and are often mentioned as an intermediate step towards completely non-invasive long-term monitoring. Many development attempts based on this proposal have been made throughout the past few decades [203–206].

Currently, there are two commercially available telemetric ICP sensors on the market [31]. They are both strain gauge micro transducers extended into the intracranial space through a burr hole. The Neurovent-P-tel (Raumedic AG, Helbrechts, Germany) is a parenchymal catheter mounted to the cranial bone. This communicates with an external telemetric reader connected to a portable data logger that displays the mean ICP scores [206, 207]. The device allows for storage of ICP recordings in a short- and a long-play mode. The short-play mode stores five mean ICP values per second (5 Hz). The long-play mode stores one ICP value per second (1 Hz); hence, long-term pulsatile monitoring was not an option. The device has been validated to provide reliable readings for a broad range of ICP values [208], and also clinically useful with minimal drift over more extended time periods than the manufacturer’s suggested 3 months [30, 209–212]. Some challenges remain, however. The chosen sampling frequency of 5 Hz is too low for adequate ICP waveform analysis [209], but sufficient for calculation of the PRx [30, 213]. In addition, the device only allows for 72 h of monitoring in the short-play mode, and the data processing software also requires improvement [207]. The loss of signal and disruption of measurements due to telemetric reader misalignment has also been reported [30].

Another telemetric ICP measurement device currently on the market is the Sensor Reservoir (Miethke, Potsdam, Germany) [207]. This system also consists of an implanted sensor and an external reader unit, but the sensor is integrated into an existing shunt system. This system has the added benefit of therapeutic shunt drainage and will reveal over or under drainage of CSF. However, long term monitoring of ICP and accurate pulsatile ICP readings are not feasible with the current device [31, 207].

### Limitations

Major challenges concerning implantable ICP micro sensors include biocompatible electronics, power sources and efficient telemetry, in addition to the issue of reference pressure drift [214]. In this study, we addressed the drift issue of current telemetric ICP devices related to mean ICP scores. A major issue is how zero reference pressures are affected, and how to control whether this is the case or not. It seems clear that the mean ICP values provided by the telemetric devices must be interpreted with caution.

Since telemetric devices are implanted, the risks of bleeds and infection are the same as for other invasive ICP measurement devices.

### Biodegradable pressure sensors

One limitation of the implantable devices described in the previous section is the need for surgical retrieval procedures, which subjects patients to the distress and risks associated with re-operation. Biodegradable sensors have therefore been explored in some detail, as they could provide added benefits because the complications are avoided. There are currently no commercially available products in this category, but technological advances have been made over the past years.

Kang et al. [215] presented a biodegradable silicon-based electronic sensor that is connected to a wireless data transmitter and potentiostat by means of wires through the skull. The pressure sensor and cables are entirely biodegradable when immersed in aqueous solutions such as CSF. The data transmitter with the potentiostat is placed under the skin (subdermally) and is not biodegradable, but because the wires through the skull are biodegradable, this can easily be removed once the pressure sensor and cables have degraded. Stable, continuous operation has been proven for up to 3 days in vivo in rats. The pressure sensor volume is 0.16 mm^3^ and is thereby small enough not to increase the ICP significantly [215]. However, very little measurement data has been provided so far.

Extending the operational lifetime of these devices is a daunting challenge in material science as the end goal is still for the sensor to be naturally eliminated and dissolved into biologically safe products [214, 216]. The easiest way to prolong the sensor’s lifetime is by using encapsulating layers, which slows down the degeneration process. The use of ultrathin films of silicon dioxide (t-SiO_2_) as encapsulation layers has been suggested as a potential solution and has been applied to biodegradable ICP sensors to increase the operational lifetime. From days 1–18, the accuracy was within ± 2 mmHg with a baseline drift within ± 1 mm Hg. On day 25, a negative drift of 4 mmHg was reported, comparable to clinical ICP monitors. After this, the signals from the device disappear, possibly due to dissolution of the sensors [217].

This field is still in its early stages, and in vivo biodegradable sensors are emerging as a powerful tool in biomedical research that has significant potential in diagnostic medicine. Clinical utility of biodegradable miniature sensors may have greater promise. While this field is also in its early phase, the focus of clinical neuroscientists should shift towards this aspect, rather than focusing on nICP approaches.

### Limitations

There are numerous challenges related to more permanent implantable pressure sensors that the field has yet to overcome. The implantable device must operate under hostile conditions. The environment within the human body is humid, filled with proteins, enzymes and ions. In addition, in vivo ICP measurements are especially challenging, because the pressure-sensitive part of the sensor must be in physical contact with the medium in which the pressure detection is being performed. Combined efforts between the scientific fields, addressing materials, biology and medicine, are therefore the most important goal going forward [218].

## Avenues for improving measurements of ICP

What are the big questions that need to be answered in this area in the coming ten or 20 years? Where should clinicians, physicists and engineers invest their time and effort to develop new technologies? How can one bring neuromonitoring into the 21st century? From our engineering and clinical perspective, we have highlighted some areas that we regard as most important. As in other fields of medicine, progress in this field is highly technology-driven. The areas for improvement presented here require technology development to lift ICP measurement practice to another level.

### Improving today’s practice of measuring mean ICP

Given that mean ICP is the most prevalent ICP score independent of measurement modality, more focus is needed on the fact that this score can be affected by the variability of reference pressure. The current ICP measurement systems lack methods for checking whether untoward changes in zero pressure level have occurred. The health care personnel are often left speculating whether the measured ICP is “real” or not. In clinical practice as well as in scientific publishing, it is crucial to know whether the mean ICP scores can be trusted.

The plotting of mean ICP scores over time is a first step toward addressing changes in reference pressure, but methodology that is more extensive is required to assess whether reference pressure changes have occurred. Means for identification of baseline pressure errors could be implemented and displayed to health care personnel during ongoing ICP monitoring. This would prevent erroneous patient management based on wrong ICP scores.

Health care personnel need to be offered better means for assessing the ICP source signal. Modern ICP measurement equipment offers, at best, the opportunity to inspect the processed ICP waveform on a vital signs monitor screen but provides limited information. Improved ICP/nICP source signal control is required for implantable ICP measuring devices and for nICP measurements. Invasive ICP measurements from dedicated ICP sensors or ventricular fluid-filled catheters would also benefit from this.

Best practice would require implementation of algorithms for automatic assessment of the ICP source signal in systems used for ICP measurements. Determining ICP scores from automatically identified single ICP waves would enhance accuracy beyond current practice. Feedback to health care personnel based on automatic ICP source signal control would reduce the impact of subjective assessments.

### Implementation of pulsatile ICP measurements as clinical routine

The current ICP monitoring practice of only measuring static ICP (mean ICP), not pulsatile ICP, implies that only parts of the information within the ICP signal are provided to the health care personnel. There are at least two reasons for measuring both static and pulsatile ICP routinely: (i) Quality control of the ICP signal, and (ii) Added information about the ICP, particularly information about ICC.

However, assessment of pulsatile ICP requires a routine for single ICP wave identification. Such algorithms are currently not included in ICP measurement devices. Without appropriate ICP wave assessment, correct pulsatile ICP assessment is not feasible. If pulsatile ICP is analyzed with more complex algorithms, the information provided to health care personnel needs to be rather straightforward. From our end, we have found assessment of the mean ICP wave amplitude (MWA) most feasible; the ICP wave rise time coefficient might be an alternative, but may be confounded by alterations in rise time.

### Prediction of events based on ICP measurements

With today’s knowledge about machine learning and artificial intelligence, digitally stored ICP measurements could be used to generate algorithms that might be able to recognize patterns in the ICP source signal that are linked to future events. Algorithms could be incorporated in clinical ICP measurement systems and thereby help health care personnel to foresee clinical events and consider treatment options to prevent clinical deterioration.

### Multi-modality monitoring

Since ICP is one among several parameters of the state of the brain, there is a need to incorporate ICP with measurements of parameters such as CBF (and autoregulatory capacity), brain oxygenation and metabolism. While the ICP provides a more global assessment of the intracranial state, several of the other technologies (CBF, oxygenation, and metabolism) provide information at the site of measurement useful for health care personel.

### Shift of attention from non-invasive technologies towards implantable ICP sensors

With regard to non-invasive ICP monitoring, there are currently no clinically useful source signals available for continuous ICP estimates. At best, single point values of ICP may be obtained. This would be useful in the pre-hospital setting, and to indicate whether abnormal ICP is present or not. However, for continuous nICP monitoring, new and yet unknown source signals need to be introduced. Given the lack of available nICP source signals, we would recommend that scientists focus more attention on developing implantable miniature pressure sensors, even biodegradable pressure sensors, utilizing wireless technologies. Given the opportunity to assess both static and pulsatile ICP, they might be placed epidurally. In addition, telemetric sensors/biodegradable sensors in the lumbar region with a focus on ICC measurements seems within reach. Long-term monitoring of ICP could become feasible. Such implantable miniature ICP sensors could become tools for surveillance of individuals with brain disease or injury, as well as in diagnostic assessment of individuals with neurological disease. At this moment, we therefore believe development of implantable miniature ICP sensors has a far greater potential than searching for means to measure nICP.

## Conclusions

Over the last 60–70 years, ICP monitoring has become an increasingly applied tool for surveillance and diagnosis of neurosurgical and neurological patients. However, the clinical benefit of ICP measurements remains a topic of debate despite a large amount of scientific literature on ICP. Class I evidence for its clinical benefit is lacking, even for TBI, which is the main area in which it is used.

One reason why the field has not moved further is limited awareness of the constraints and weaknesses of the current ICP measurement approaches. Clinical practice and scientific reporting is thereby hampered by uncertainty about the ICP scores. In this critical review, we have addressed two areas in particular: the role of ICP source signal control and the impact of reference pressure variability on static ICP scores. For a number of reasons, these issues have received less attention in the past, not least because ICP monitoring systems provide limited opportunities for exploring these methodological weaknesses. In our opinion, the major issue with today’s practice is lack of signal quality control. As long as these issues remain largely unexplored, they cannot be defined as clinically irrelevant. All issues that may erroneously alter the ICP scores provided to health care personnel are important, as incorrect ICP scores can result in wrong treatment. These aspects deserve greater examination from the ICP community. The field of ICP measurements needs to take a step from analog to digital technology.

The limitations in ICP source signal quality control and reference pressure variability become even more important as new technologies, including implantable ICP micro sensors for long-term ICP measurements, enter the market. With regard to nICP measurements, adequate control of nICP source signals is a requirement.

Finally, it should be remembered that measurement of ICP is not a treatment per se; it is one modality for surveillance and diagnostics of the brain that in turn may aid in defining the best medical or surgical treatment. It seems evident, for a physiological monitoring modality to have the best possible clinical utility, that in-depth knowledge about the limitations and weaknesses of the modality is required.

## Supplementary information

**Additional file 1: Movie 1.** A continuous ICP signal. A continuous ICP signal is pulsatile and characterized by the pressure changes occurring during each cardiac beat (illustrated by the light blue line). During each cardiac contraction the ICP increases from a diastolic minimum pressure (illustrated by red filled circle) to a systolic maximum pressure (illustrated dark blue X). The mean ICP is given by the dark blue line, and represents the average pressure of all data points, relative to a reference pressure, i.e. the pressure sensor is zeroed against the atmospheric pressure before being inserted to the intracranial compartment.

**Additional file 2: Movie 2.** A continuous ICP signal is highly dynamic. Online monitoring of ICP reveals that the ICP signal is highly dynamic. Each image lasts 6 s and demonstrates variation over time for both the ICP scores mean ICP (Current static pressure) and for the mean ICP wave amplitude (MeanWaveAmp).

**Additional file 3: Movie 3.** Continuous ICP signals from two ICP sensors placed nearby in the brain parenchyma reveals constant shift in baseline reference pressure. The continuous ICP signals, characterized by diastolic minimum (red dots) and systolic maximum (blue x) pressures, from two different Codman ICP sensors placed nearby in the brain, wherein the mean ICP (dark blue lines) is diverging while the ICP waveforms of the two signals are close to identical.

**Additional file 4: Movie 4.** Continuous ICP signals from two ICP sensors placed nearby in the brain parenchyma reveals a sudden shift in baseline reference pressure. The continuous ICP signals from two different Codman ICP sensors placed nearby in the brain show a sudden shift in baseline reference pressure of one of the ICP sensors. The mean ICP (dark blue lines) is suddenly diverging while the ICP waveforms of the two signals remain close to identical.

## Data Availability

Data sharing is not applicable to this article as no datasets were generated during the current study. The ICP recording presented in Fig. 4 is available upon request.

## Abbreviations

AAMI: Association for the Advancement of Medical Instrumentation
AMP: Amplitude
ANSI: The American National Standards Institute
BP: Blood pressure
BPE: Baseline pressure error
CBF: Cerebral blood flow
CBFV: Cerebral blood flow velocity
CNS: Central nervous system
CPP: Cerebral perfusion pressure
CSF: Cerebrospinal fluid
CT: Computed tomography
dP: Delta pressure
dV: Delta volume
ESD: Electrostatic discharge
IAAC: Intracranial arterial amplitude correlation
ICE: Intracranial elastance
ICC: Intracranial compliance
ICP: Intracranial pressure
ICU: Intensive care unit
IIH: Idiopathic intracranial hypertension
iNPH: Idiopathic normal pressure hydrocephalus
LP: Lumbar puncture
nICP: Non-invasive ICP
mmHg: Millimeter mercury
MOCAIP: Morphological Clustering and Analysis of ICP pulse
MRI: Magnetic resonance imaging
MWA: Mean wave amplitude
NIRS: Near infrared spectroscopy
ONSD: Optic nerve sheath diameter
PbtO_2_: Brain tissue oxygenation
Pa: Pascal
P1: Systolic peak
P2: Tidal peak
P3: Dicrotic peak
PI: Pulsatility index
PRx: Pressure reacitivity index
PVI: Pressure–volume index
RAP: correlation coefficient between AMP and mean ICP
RCT: Randomized controlled trial
SAH: Subarachnoid hemorrhage
TBI: Traumatic brain injury
TCA: Transcranial acoustic signals
TCD: Transcranial Doppler
TOF: Time of flight
VPR: Volume–pressure response
VPT: Volume–pressure test
