# Supplementary figures and images for: Measuring intracranial pressure by invasive, less invasive or non-invasive means: limitations and avenues for improvement
Source: Fluids Barriers CNS. 2020 May 6;17:34. doi: 10.1186/s12987-020-00195-3 (PMC7201553; doi:10.1186/s12987-020-00195-3)

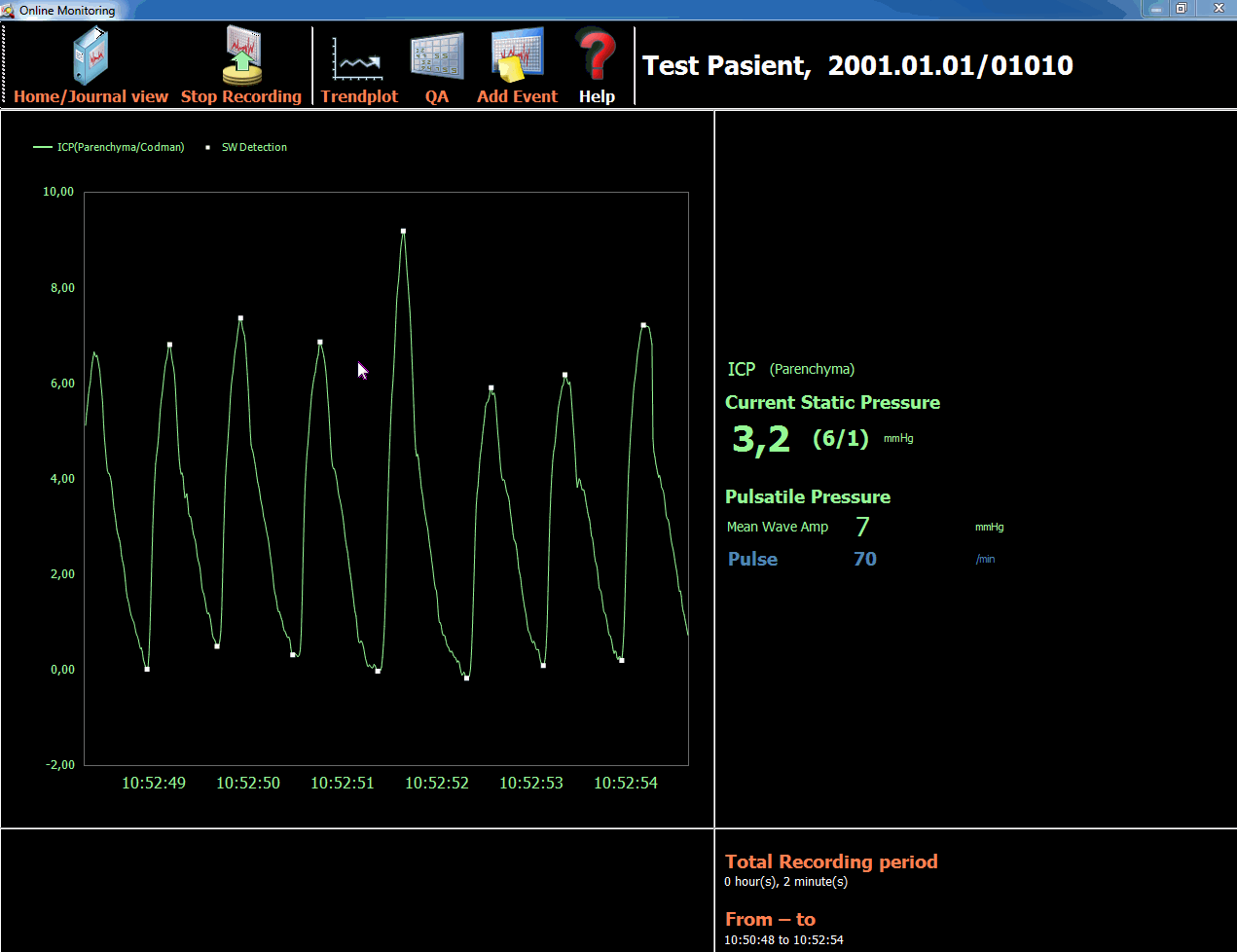

Supplement: Supplementary file 2 — Additional file 2: Movie 2. A continuous ICP signal is highly dynamic. Online monitoring of ICP reveals that the ICP signal is highly dynamic. Each image lasts 6 s and demonstrates variation over time for both the ICP scores mean ICP (Current static pressure) and for the mean ICP wave amplitude (MeanWaveAmp). [file 12987_2020_195_MOESM2_ESM.gif]
